# Enantioenriched Positive Allosteric Modulators Display Distinct Pharmacology at the Dopamine D_1_ Receptor

**DOI:** 10.3390/molecules26133799

**Published:** 2021-06-22

**Authors:** Tim J. Fyfe, Peter J. Scammells, J. Robert Lane, Ben Capuano

**Affiliations:** 1 Medicinal Chemistry, Monash Institute of Pharmaceutical Sciences, Monash University, 381 Royal Parade, Parkville, VIC 3052, Australia; tim.fyfe@griffithhack.com (T.J.F.); peter.scammells@monash.edu (P.J.S.); 2 Drug Discovery Biology, Monash Institute of Pharmaceutical Sciences, Monash University, 381 Royal Parade, Parkville, VIC 3052, Australia; 3 School of Life Sciences, Queen’s Medical Centre, University of Nottingham, Nottingham NG7 2UH, UK

**Keywords:** dopamine D_1_ receptor, positive allosteric modulator, PAM, G protein-coupled receptors, synthetic medicinal chemistry, pharmacological evaluation, enantioenriched, chiral auxiliary, cAMP BRET

## Abstract

(1) Background: Two first-in-class racemic dopamine D_1_ receptor (D_1_R) positive allosteric modulator (PAM) chemotypes (**1** and **2**) were identified from a high-throughput screen. In particular, due to its selectivity for the D_1_R and reported lack of intrinsic activity, compound **2** shows promise as a starting point toward the development of small molecule allosteric modulators to ameliorate the cognitive deficits associated with some neuropsychiatric disease states; (2) Methods: Herein, we describe the enantioenrichment of optical isomers of **2** using chiral auxiliaries derived from (*R*)- and (*S*)-3-hydroxy-4,4-dimethyldihydrofuran-2(3*H*)-one (d- and l-pantolactone, respectively); (3) Results: We confirm both the racemate and enantiomers of **2** are active and selective for the D_1_R, but that the respective stereoisomers show a significant difference in their affinity and magnitude of positive allosteric cooperativity with dopamine; (4) Conclusions: These data warrant further investigation of asymmetric syntheses of optically pure analogues of **2** for the development of D_1_R PAMs with superior allosteric properties.

## 1. Introduction

Schizophrenia (SCZ) is a debilitating neuropsychiatric illness characterised by three distinct symptoms domains [1,2]. Positive symptoms describe manifestations of psychosis such as delusions, whereas negative symptoms are defined as alterations in drive and volition [3]. Cognitive deficits are a central feature of SCZ, and include deficits in working memory, attention, learning, and executive functioning [4]. Numerous studies have demonstrated an association between the severity of cognitive impairment and functional, social, and occupational outcomes in SCZ. Thus, the development of therapeutics that address this symptom domain are desirable [5]. Unfortunately, current clinical antipsychotic drugs (APDs) fail to address these cognitive symptoms [6,7].

Hypodopaminergic function in the dorsolateral prefrontal cortex (dlPFC), an area associated with cognitive control and executive functions including working memory and selective attention [8], is thought to be related to negative symptoms and cognitive deficits [9,10]. Indeed, both dopamine receptor (DR) antagonists and dopamine (DA) depletion in the dlPFC impair cognitive function [11,12,13]. There is mounting evidence to suggest that D_1_R agonists can reverse these deficits [14,15], although excessive D_1_R stimulation may impede cognitive function [16,17]. Many orthosteric D_1_R agonists that compete with DA for the orthosteric binding site display a lack of subtype selectivity (relative to other DRs) as well as poor pharmacokinetic properties. The benzazepine class of D_1_R agonists have poor bioavailability [18] and has the propensity to lower seizure thresholds [19]. Similarly, the D_1_R agonist dihydrexidine displays poor oral bioavailability and is rapidly metabolised in vivo [20]. Orthosteric D_1_R agonists have also been shown to increase incidence of drug-induced hypotension, potentially mediated by D_1_Rs expressed in the periphery [21], as well as a rapid acquisition of tolerance [22,23]. There is clearly an unmet need for the development of selective, bioavailable small molecule D_1_R ligands to further interrogate their potential utility to treat cognitive deficits associated with SCZ.

There has been tremendous recent progress in the development of novel D_1_R positive allosteric modulators (PAMs) [24,25,26,27,28,29] in concert with X-ray crystal [30] and cryo-EM structures [31,32] that have revealed fascinating structural insights into the therapeutic potential of the D_1_R. This has culminated in the clinical evaluation of the first D_1_R PAM LY3154207 (Mevidalen) [33] which is currently in phase 2 for the amelioration of cognition associated with Lewy body dementias. PAMs represent an alternative approach to targeting the D_1_R and act to modulate the affinity and/or efficacy of DA from a topographically distinct but conformationally linked binding site. The engagement of a less conserved allosteric binding site may confer greater subtype selectivity than orthosteric D_1_R agonists. A D_1_R PAM that displays positive allosteric cooperativity but lacks intrinsic efficacy which, in its own right, might maintain the temporal and spatial patterns of DA neurotransmission [34,35].

**Figure 1:**
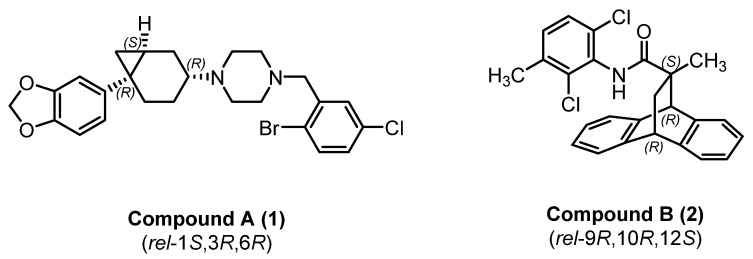
High-throughput screening hits racemic Compound A [1-((*rel*-1*S*,3*R*,6*R*)-6-(benzo[*d*][1,3]dioxol-5-yl)bicyclo [4.1.0]heptan-3-yl)-4-(2-bromo-5-chlorobenzyl)piperazine] (1) and racemic Compound B [*rel*-(9*R*,10*R*,12*S*)-*N*-(2,6-dichloro-3-methylphenyl)-12-methyl-9,10-dihydro-9,10-ethanoanthracene-12-carboxamide] (*rac*-**2**) reported by Lewis et al. [36].

Lewis et al. recently identified two racemic D_1_R PAM chemotypes (Compound A, (**1**) and Compound B, (*rac*-**2**), Figure 1) [36]. **1** was shown to be a D_1_R PAM but also acted as an agonist at the D_2_R and thus was not investigated further. As D_2_R agonism is known to exacerbate the positive symptoms of SCZ, achieving selectivity for the D_1_R versus the D_2_R is paramount for the development of efficacious cognitive-enhancing therapeutics. *rac*-**2** was shown to have superior potency compared to **1** whilst being selective for the human D_1_R. To our knowledge there is no reported chemical synthesis and biological characterisation of optical isomers of **2**. Herein, we report the synthesis and pharmacological characterisation of (*rac*)-**2**, and enantioenriched optical isomers of **2** (herein denoted as (*S*)-**2** and (*R*)-**2**). These enantioenriched samples were accessed by employing various chiral auxiliaries in an asymmetric Diels Alder (ADA) cycloaddition reaction.

To characterise their pharmacology, we applied analytical pharmacological methods to determine allosteric ligand affinity for the unoccupied receptor, the strength of modulatory effects upon DA activity as well as the magnitude of allosteric agonism [37]. We demonstrate that enantiomers of **2** can be accessed in moderate enantiopurity in four synthetic steps. Importantly we reveal that these enantiomers display different affinities for the D_1_R as well as different levels of positive cooperativity with DA. The degree of allosteric cooperativity required for a ‘clinical’ D_1_R PAM is currently unknown. These data illustrate the importance of characterising optically pure analogues for future structure-activity relationship studies of **2**.

## 2. Results and Discussions

### 2.1. Chemistry

**Scheme 1:**
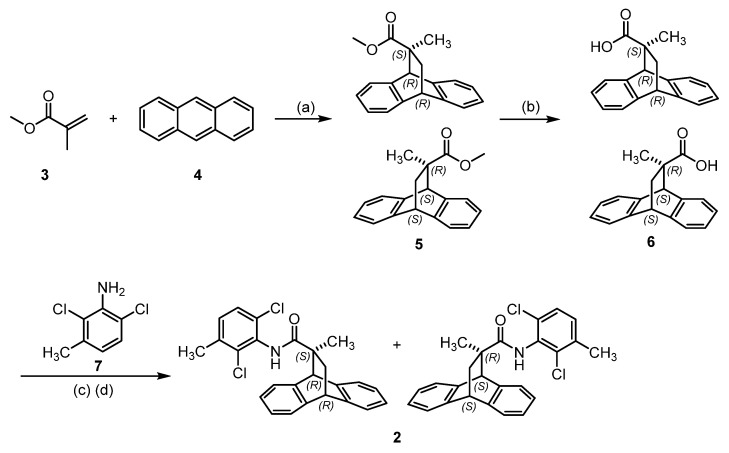
Chemical synthesis of *rac*-**2**. *Reagents and conditions*: (**a**) AlCl_3_, CH_2_Cl_2_, r.t. 72 h, 63%; (**b**) NaOH, 1:1 THF/H_2_O, reflux 7 days, 98%; (**c**) SOCl_2_, DMF_(cat.)_, reflux, 24 h; (**d**) DMAP_(cat.)_ DIPEA, MeCN, reflux, 24 h, 25%.

Compound *rac*-**2** was resynthesised in three steps according to Scheme 1 [38]. Firstly, AlCl_3_-catalysed Diels-Alder [4 + 2]cycloaddition of methyl methacrylate **3** with anthracene **4** afforded the 9,10 bridged ester **5** as a racemic mixture. Ester saponification was achieved under forcing conditions with NaOH in THF/H_2_O at reflux, affording acid **6**. Subsequent treatment with thionyl chloride and DMF, followed by DMAP-catalysed nucleophilic substitution with commercially available 2,6-dichloro-3-methylaniline (**7**) afforded *rac*-**2**. High-performance liquid chromatography (HPLC) using an amylose chiral stationary phase (CSP) verified the presence of two enantiomers (see Supplementary Materials).

**Scheme 2:**
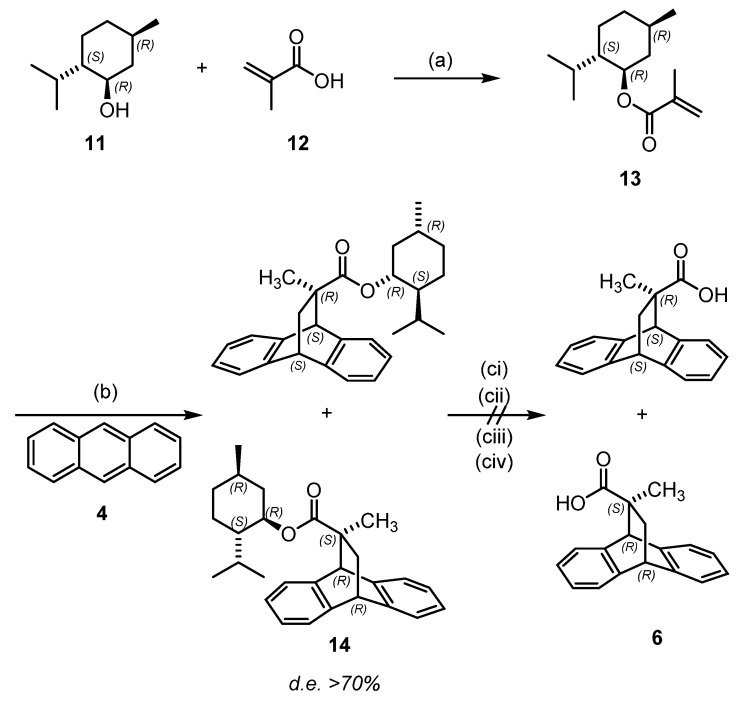
Chemical synthesis of menthol-derived diastereomers using ADA chemistry. Reagents and conditions: (**a**) EDCI, DMAP _(cat.)_, anhydrous CH_2_Cl_2_, r.t. 48 h, 55%; (**b**) TiCl_4_, dry CH_2_Cl_2_, r.t. 15 h, 25%; (**ci**) NaOH, THF/H_2_O, reflux 7 d; (**cii**) H_2_SO_4_ (conc.), 1,4-dioxane, reflux, 3 h; (**ciii**) NaOH, 2:1:1 H_2_O, MeOH, *t*-butanol, reflux 72 h; (**civ**) FeCl_3_·6H_2_O, 1:4 1,4-dioxane/H_2_O, reflux.

It is not clear from the current literature regarding the newly identified D_1_R PAM scaffold whether a single enantiomer or racemic mixture is responsible for the quoted in vitro activity. This led us to investigate methods to generate optically pure stereoisomers of compound **2**. Numerous attempts at chiral resolution of **5** (semi-preparative chiral HPLC) and the chromatographic resolution of derivatives of **5** (diastereomeric amides/esters, diastereomeric salts) using various resolving ligands, e.g., (*R*)-1-phenylethan-1-amine, (*S*)-2-amino-2-phenylethan-1-ol, and (1*R*,2*S*,5*R*)-2-isopropyl-5-methylcyclohexan-1-ol ((−)-menthol)), all failed. Therefore, to access enantiopure synthetic precursors, it was necessary to develop and explore the use of chiral auxiliaries in an ADA reaction. The Diels-Alder reaction arises from high regio- and stereoselectivity which also entails the use of various functionalised dienes and dienophiles, and much work has been done in the last three decades to develop ADA reactions, based on both enantiopure dienes and enantiopure dienophiles [39]. Accordingly, the area has been extensively reviewed [40,41,42].

(−)-Menthol **11**, a chiral molecule with three stereocentres, was initially selected as a practical chiral auxiliary to probe the diastereoselectivity of the Diels-Alder cycloaddition. A derivative of **11**, (−)-8-phenylmenthol, has previously been shown to induce ADA reactions with high distereoselectivity under AlCl_3_ catalysis [43]. As outlined in Scheme 2, DMAP-catalysed, EDC-mediated Steglich-type [44] esterification of methacrylic acid **12** with **11** furnished the corresponding auxiliary **13**. This dienophile was subjected to a TiCl_4_-catalysed [4 + 2]cycloaddition, affording cycloadduct(s) **14**. ^1^H NMR analysis confirmed that the (1*R*,2*S*,5*R*)-menthol-acrylate chiral auxiliary conferred moderate diastereoselectivity in the cycloaddition as one diastereomer could be identified as the major product (>70% d.e.). Unfortunately, hydrolytic ester cleavage of **14** using LiOH or NaOH in refluxing THF/H_2_O failed to furnish the acid (**6**) as no reaction was evident via LC/MS analysis. Alternative conditions for hydrolysis were sourced from work completed by Myers et al., who reported the use of various synthetic examples and reagents and to hydrolyse amides and esters [45]. Such additional methods were explored, including stirring **14** at reflux in the presence H_2_SO_4_ and 1,4-dioxane, stirring **14** reflux in 2:1:1 solution of H_2_O, MeOH and *tert*-butanol in the presence of NaOH, as well as at reflux in a 4:1 solution of H_2_O/1,4-dioxane in the presence of the Lewis acid iron(III) chloride hexahydrate. These chemistries all failed to provide the target acid.

**Scheme 3:**
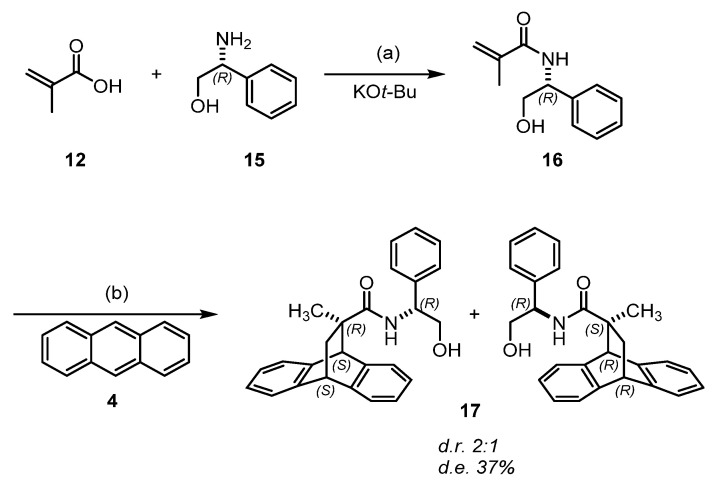
Chemical synthesis of 2-phenylglycinol ethanoanthracene diastereomers. Reagents and conditions: (**a**) dry THF, r.t., 2.5 h, 91%; (**b**) TiCl_4_, dry DCM, r.t. 7 days, 75%.

An additional auxiliary, (*R*)-2-amino-2-phenylethan-1-ol **15**, was investigated for its potential in ADA chemistry. According to Scheme 3, methacrylic acid **12** was subjected to a potassium *tert*-butoxide mediated direct amidation [46] using **15**, yielding the corresponding acrylamide **16**. Again, the newly formed acrylamide was directly utilised in a TiCl_4_-cataysed [4 + 2]cycloaddition to furnish the desired ethanoanthracene derivative(s) **17** as a beige solid. HPLC analysis of the purified material indicated the presence of two diastereomers in an approximate diastereomic ratio (3:1) and this was further confirmed with ^1^H NMR spectroscopy (~37% d.e.).

The study of ADA induction involving esters derived from achiral acrylic and methacrylic acids, and the chiral auxiliaries (*R*)- and (*S*)-3-hydroxy-4,4-dimethyl-1-phenyl-2-pyrrolidinone with different dienes including anthracene (which was of particular interest), catalysed by TiCl_4_ has been described [39]. These studies reported adducts derived from anthracene and the (*R*)-pantolactam-acrylate ester could be obtained with high facial-diasteroselectivity where subsequent ester saponification afforded the corresponding enantiopure acid in high optical purity. In addition, the acrylates of (*R*)-pantolactone have also been extensively studied for their asymmetric capacity to direct cycloadditions with high facial-diastereoselectivity for a number of diene scaffolds under TiCl_4_ catalysis, including anthracene [47,48,49]. Pantolactones and their use as chiral auxiliaries in ADA chemistry has been extensively reviewed [50], as well as mechanistic models proposed to explain the sense of asymmetric induction in TiCl_4_-catalysed ADA reactions of pantolactone-acrylate derivatives [51].

**Scheme 4:**
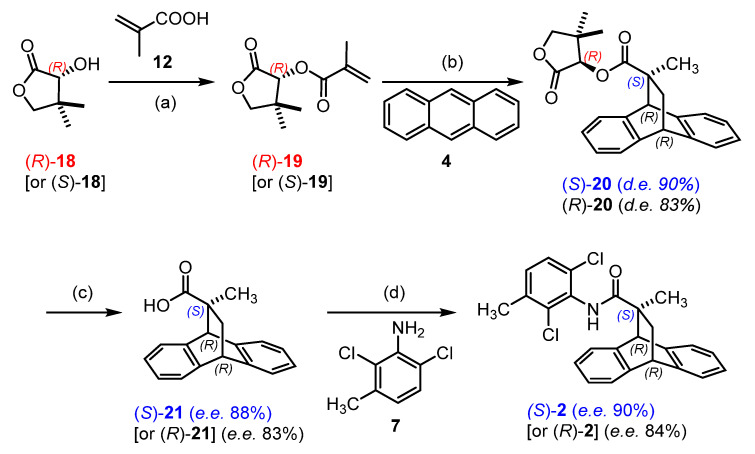
Enantioenrichment of optical isomers of **2** using chiral auxiliaries derived from pantolactone. Reagents and conditions: (**a**) EDCI, DMAP, dry CH_2_Cl_2_, r.t. 10 h, 69–87% ((*R*)-**19**, (*S*)-**19**); (**b**) TiCl_4_, dry CH_2_Cl_2_, r.t. 15 h, 80–85% ((*S*)-**20**, (*R*)-**20**); (**c**) NaOH, THF/H_2_O, reflux, 72 h, 85–98% ((*S*)-**21**, (*R*)-**21**); (**d**) SOCl_2 (neat)_, DMF_(cat.)_ reflux, 24 h, then acyl halide, DMAP_(cat.)_ DIPEA, MeCN, reflux, 24 h, 19–25% ((*S*)-**2**, (*R*)-**2**).

Following an adapted procedure from Camps et al. [39], and as outlined in Scheme 4, commercially available d-pantolactone ((*R*)-**18**) was reacted with methacrylic acid **12** under modified Steglich esterification [44] conditions to give the corresponding auxiliary (*R*)-**19** in moderate yield. Subsequent [4 + 2] cycloaddition with anthracene **4** as the diene in the presence of TiCl_4_ as catalyst, followed by chromatographic purification and recrystallization from EtOH, afforded (*S*)-**20** as a pale white crystalline solid. ^1^H NMR spectroscopic analysis confirmed high diasteroselectivity (>90% d.e.) for the asymmetric induction. Basic ester saponification using a large excess of NaOH in refluxing THF/H_2_O was achieved to eventually yield carboxylic acid (*S*)-**21**. Although the synthesis of ((*S*)-**21**) has been reported in the literature using (*R*)-3-hydroxy-4,4-dimethyl-1-phenyl-2-pyrrolidinone as a chiral auxiliary [39] our synthesis was performed to examine the asymmetric induction potential of pantolactone chiral auxiliaries. Moreover, ((*S*)-**21**) was subsequently required to form the corresponding amide (*S*)-**2**, so as it could be evaluated for its in vitro allosteric ligand parameters. The enantiopurity of (*S*)-**21** was characterised using polarimetry [α]_D_^25^ = −24.9° (*c* 1.0, CHCl_3_) which was in accordance with literature values [39]. Based on approximations from the preceding ^1^H NMR spectrum of (*S*)-**20**, an enantiomeric excess (e.e.) of >90% was likely. Indeed, chiral HPLC (cHPLC) confirmed the presence of predominantly a single enantiomer, supporting the high facial-diastereoselectivity of the pantolactone chiral auxiliary in [4 + 2] cycloadditions, with e.e. calculated to be ~88%. The enantioenriched mixture was successively activated with thionyl chloride and subjected to nucleophilic substitution employing conditions outlined previously, yielding enantioenriched (*S*)-**2**. CHPLC analysis of (*S*)-**2** determined the e.e. to be ~90%.

Interestingly, Camps et al. reported that a racemic *N*-phenyl pantolactam chiral auxiliary was unable to react with anthracene under AlCl_3_ catalysis [39]. In addition, the authors did not report on the effectiveness of TiCl_4_ as catalyst for the cycloaddition reaction between anthracene and their (*S*)-*N*-phenyl pantolactam chiral auxiliary, nor its racemic counterpart. Thus, the asymmetric induction potential of using the structurally related (*S*)-pantolactone acrylate ester ((*S*)-**19**) as an alternative chiral auxiliary to access the previously unreported (*R*)-**20** was unknown. This chemistry is illustrated in Scheme 4 commencing with Steglich esterification [44] of **12** with commercially available l-pantolactone (*S*)-**18**) using conditions outlined previously to afford (*S*)-**19**. Subsequent cycloaddition of the auxiliary (*S*)-**19** with diene **4** gave cycloadduct (*R*)-**20** in good yield, with ^1^H NMR indicating a slightly lower diastereoselectivity (d.e. ~83%). This apparent reduced asymmetry may have arisen from a failure for (*R*)-**20** to recrystallize, or is mechanistically inherent in the cycloaddition of (*S*)-**19**. Hydrolytic cleavage of the auxiliary was achieved using forcing alkaline conditions, eventually affording the free carboxylic acid (*R*)-**21**. An assessment of the enantiopurity was again made using polarimetry ([α]_D_^25^ = +24.1° (*c* 1.0, CHCl_3_)), whereby subsequent chiral HPLC analysis determined the e.e. to be ~82%. Acyl halide formation and nucleophilic displacement with **7** using conditions outlined previously furnished the corresponding enantioenriched (*R*)-**2**. CHPLC analysis of (*R*)-**2** determined the e.e. to be ~84%.

### 2.2. Pharmacology

**Figure 2:**
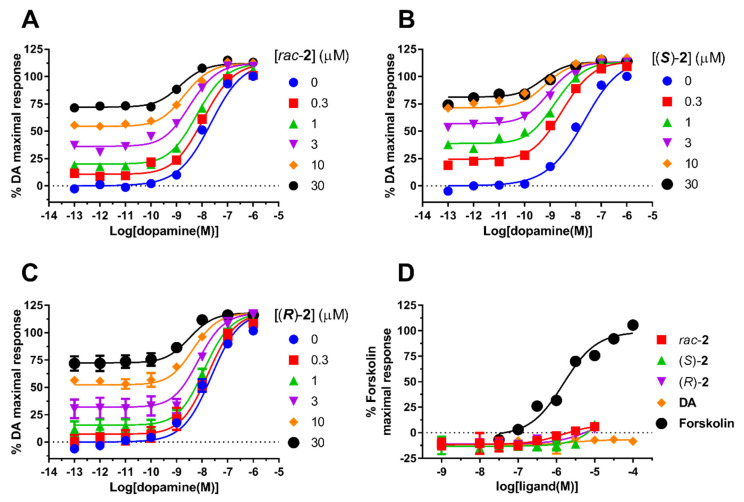
All compounds display allosteric pharmacology at the D_1_R in an assay measuring cAMP production using a BRET biosensor. *rac*-**2** (**A**), the enantioenriched *S*-isomer (*S*)-**2**, (**B**), and the enantioenriched *R*-isomer (*R*)-**2**, (**C**) act as an ago-PAMs, potentiating DA potency and exerting allosteric agonism. These data were fitted to an operational model of agonism to derive values of affinity, cooperativity with DA and intrinsic efficacy (see Table 1). Data are presented as mean ± S.D. from at least four separate experiments. (**D**) All compounds were assessed for their activity at non-hD_1_R/hD_2_R expressing FlpIn CHO-cells transfected with the BRET biosensor, demonstrating that compound activity is mediated through the D_1_/D_2_Rs. Data are presented as mean ± S.D. from at least four separate experiments.

Previous work by Lewis et al. [36] reported *rac*-**2** as a PAM at the D_1_R with superior potency (EC_50_ = 43 nM) and no agonist activity. We tested *rac*-**2** in an assay measuring accumulation of cyclic adenosine monophosphate (cAMP) through activation of the hD_1_R stably expressed in FlpIn CHO cells using a BRET biosensor [52]. Concentration-response curves of DA were generated in the presence of increasing concentrations of test compound. An operational model of allostery was applied to these data, allowing us to determine estimates of functional affinity for the unoccupied receptor (*K_B_*), a composite measure of allosteric cooperativity (*αβ*) that combines cooperativity with DA affinity (*α*) and modulatory effect upon DA efficacy (*β*), as well as the intrinsic efficacy (*τ_B_*) of the allosteric ligand. Values of *αβ* > 1 indicate a positive modulatory effect. Functional assessment of *rac*-**2** in our hands showed it exhibited modest affinity for the D_1_R (*K_B_* = 1.6 µM, Figure 2A, Table 1), acting to potentiate the potency of DA 100-fold (*αβ* = 100) as well as display allosteric agonism (*τ_B_* = 2.1, Table 1). Lewis and colleagues observed that *rac*-**2** did not display agonism. This difference likely reflects a difference in the cell background (CHO versus HEK293) and/or a higher level of D_1_R expression in our cell line and/or a greater sensitivity of our cAMP assay [36]. Indeed, such effects have been observed for a PAM of the muscarinic M_1_ acetylcholine receptor and such observations are consistent with a two-state model of allostery [53]. Consistent with the above differences, the potency of DA in our assay is also greater than that observed by Lewis and coworkers in their experiments. Note that Lewis et al. also determined the value of potency for *rac*-**2** as 40 nM. This value was determined by the measuring the potency with which *rac*-**2** causes a shift in DA potency and cannot be directly compared with the value of *K_B_* determined in our analysis that reflects the affinity of the PAM for the unoccupied receptor.

Intriguingly, the optical isomers of *rac*-**2** ((*S*)-**2**, (*R*)-**2**) were shown to display significant differences both in their affinity for the D_1_R and their degree of positive allosteric cooperativity with DA. The enantioenriched (*S*)-**2** was determined to have comparable functional affinity, efficacy and cooperativity as compared to *rac*-**2** (*K_B_* = 1 µM, *αβ* = 125, *τ_B_* = 2.5) (Figure 2B, Table 1). Conversely, the enantioenriched (*R*)-**2** displayed 4.8-fold and 7.4-fold lower functional affinity (*K_B_* = 7.4 µM *αβ* = 31) and 3-fold and 4-fold lower cooperativity with DA, relative to *rac*-**2** and (***S***)-**2** respectively (Figure 2C, Table 1). It is interesting to note that the pharmacology of *rac*-**2** is not significantly different from (*S*)-**2**. This can be reconciled by the lower levels of affinity and cooperativity with DA that (*R*)-**2** displays. These two factors combined mean that (*S*)-**2** would effectively display a 30-fold greater affinity for the DA occupied receptor as compared to (*R*)-**2** even though their affinities for the unoccupied are a more modest 7-fold different. In the racemate, therefore, (*S*)-**2** would be expected to dominate meaning that the pharmacology of the racemate reflects this enantiomer. We extended our functional characterisation of these compounds to an assay measuring inhibition of forskolin-stimulated cAMP accumulation in hD_2L_R-expressing FlpIN CHO cells. *rac*-**2** and its respective enantiomers ((*R*)-**2**, (*S*)-**2**) displayed no activity at the D_2_R up to a measured concentration of 30 µM. None of the above compounds displayed any off-target response in FlpIn CHO cells transfected with the BRET biosensor, but not expressing either the D_1_R or the D_2_R (Figure 2D). Together our data indicate that the distinct structural configuration of the two enantiomers of **2** confer differences in their ability to potentiate DA at the D_1_R.

## 3. Experimental

### 3.1. General

Solvents and fine chemicals were commercially available from standard suppliers and used where described without further purification. Davisil silica gel (40–63 μm) for Flash column chromatography used Davisil silica gel (40–63 μm) and was supplied by Grace Davison Discovery Sciences (Victoria, Australia). Deuterated solvents for NMR spectroscopy were sourced by Novachem Pty. Ltd., Victoria, Australia (a distributor for Cambridge Isotope Laboratories, Inc., Tewksbury, MA, USA). Reactions were monitored by thin layer chromatography on commercially available precoated aluminum-backed plates (Merck Kieselgel 60 F_254_). Visualization was by inspection under UV light (254 nm/366 nm). Ninhydrin (in EtOH) was used to visualize primary and secondary amines. Anhydrous Na_2_SO_4_ was used to dry all organic extracts collected after aqueous workup procedures before gravity/vacuum filtration and evaporation to dryness. Organic solvents were removed under reduced pressure at a water bath temperature of ≤40 °C. ^1^H NMR and ^13^C NMR spectra were recorded on a Bruker Avance Nanobay III 400 MHz Ultrashield Plus spectrometer at 400.13 and 100.62 MHz, respectively. Chemical shifts (δ) are recorded in parts per million (ppm) with reference to the chemical shift of the deuterated solvent. Coupling constants (*J*) are recorded in Hz, and multiplicities described by singlet (s), doublet (d), triplet (t), quadruplet (q), broad (br), multiplet (m), doublet of doublets (dd), and doublet of triplets (dt). All NMR experiments were performed in CDCl_3_ for comparison of spectra of various analogues. For analogues that displayed reduced solubility in CDCl_3_ then experiments were performed in CD_3_OD, DMSO-*d*_6_, or acetone-*d*_6_. LCMS experiments -System A (default): an Agilent 6100 series single quad coupled to an Agilent 1200 series HPLC instrument using the following buffers: buffer A, 0.1% HCOOH in H_2_O; buffer B, 0.1% HCOOH in CH_3_CN. The following gradient was used with a Phenomenex Luna 3 μm C8(2) 15 × 4.6 mm column, flow rate of 0.5 mL/min, total run time of 12 min: 0–4 min 95% buffer A/5% buffer B, 4–7 min 0% buffer A/100% buffer B, 7–12 min 95% buffer A/5% buffer B. Mass spectra were acquired in both negative & positive ion modes with a scan range of 0–1000 *m*/*z* at 5 V. UV detection was monitored at 254 nm. System B: an Agilent 6120 series single quad coupled to an Agilent 1260 series HPLC instrument using the following buffers; buffer A, 0.1% HCOOH in H_2_O; buffer B, 0.1% HCOOH in CH_3_CN. The following gradient was used with a Poroshell 120 EC-C18 50 × 3.0 mm, 2.7 μm column, flow rate of 0.5 mL/min, total run time of 5 min: 0–1 min 95% buffer A/5% buffer B, 1 to 2.5 min 0% buffer A/100% buffer B, held until 3.8 min, 3.8–4 min 95% buffer A/5% buffer B, held until 5 min. Mass spectra were acquired in both negative & positive ion modes with a scan range of 100–1000 *m*/*z*. UV detection was monitored at 214 and 254 nm. All retention times (*t*_R_) were quoted in minutes. System C: analytical reverse-phase HPLC was performed, to verify purity, on a Waters HPLC system coupled directly to a photodiode array detector and fitted with a Phenomenex Luna C8 (2) 100 Å column (150 × 4.6 mm, 5 μm). A binary solvent system of buffer A, 0.1% TFA/H_2_O; buffer B, 0.1% TFA/80% CH_3_CN/H_2_O was used. Gradient elution was attained using 100% buffer A to 100% buffer B over 12 min at a flow rate of 1 mL/min. All target compounds subjected to biological testing were >95% pure by HPLC at two wavelengths (214 and 254 nm). Analytical chiral-HPLC were conducted on an Agilent Infinity 1260 system fitted with either one of (a) Lux 5 μm Amylose-2 150 × 4.60 mm, or (b) Lux 5 μm Cellulose-1 150 × 4.60 mm. A binary solvent system was used (solvent A: ethanol; solvent B: petroleum spirits), with UV detection at 254 nm. The method used isocratic elution of 1–20% solvent A and 99–80% solvent B, with a flow rate of 1 mL/min. Mass spectra were acquired in in both negative & positive ion mode with a scan range of 100–1000 *m*/*z*. UV detection was monitored at 214 and 254 nm with retention times (*t*_R_) expressed in minutes. All screening compounds displayed >95% purity unless otherwise specified in the individual monologue.

### 3.2. Chemistry

#### 3.2.1. General Procedure A for Steglich Esterification

A mixture of methacrylic acid (1.0 equiv.) alcohol (1.0 equiv.), EDC (1.1 equiv.) and DMAP (5 mol %) in dry DCM was stirred at rt until complete consumption of the starting acid or alcohol. The reaction mixture was washed with sat. aqueous citric acid (3 × 50 mL) and sat. aqueous NaHCO_3_ (3 × 50 mL), dried (Na_2_SO_4_) and concentrated in vacuo. The residue was purified by flash column chromatography (FCC) with an appropriate eluent as indicated.

#### 3.2.2. General Procedure B for TiCl_4_-Catalysed Diels-Alder Cycloaddition

A solution of TiCl_4_ (2.0 equiv.) in anhydrous DCM (20 mL) was added to a solution of the appropriate chiral auxiliary/dienophile (1.0 equiv.) in anhydrous DCM (30 mL), and the mixture stirred at rt for 15 min. Then, a solution of the anthracene (1.0 equiv.) in dry CH_2_Cl_2_ (20 mL) was added and the mixture stirred at r.t. for 15 h. A small amount of H_2_O was added to destroy the TiCl_4_ complexes, the mixture was filtered and the filtrate was dried with anhydrous Na_2_SO_4_. The filtrate was concentrated in vacuo and the residue was purified by FCC using an appropriate eluent as indicated.

#### 3.2.3. General Procedure C for Alkaline Ester Hydrolysis

To a solution of ester (1.0 equiv.) in a 1:1 mixture of THF/H_2_O was added NaOH (3 equiv.), and the mixture stirred at reflux temperature until complete consumption of the starting material was evident. The THF was removed in vacuo and the remaining aqueous phase was washed with Et_2_O. The aqueous phase was then acidified (pH = 1), and any precipitated carboxylic acid was collected via vacuum filtration and recrystallised from EtOAc/PE. Similarly, the aqueous phase could be extracted with DCM and the organic extracts dried (Na_2_SO_4_) to afford the desired carboxylic acid to maximise the yield.

#### 3.2.4. General Procedure D for Acyl Halide Formation and Nucleophilic Substitution

The carboxylic acid (1 equiv.) was taken up in thionyl chloride (5 mL) followed by three drops of DMF and the reaction was stirred at reflux temperature until complete consumption of the starting material. The solvent was removed in vacuo and the residue was taken up in dry MeCN (25 mL). To this solution was added 2,6-dichloro-3-methylaniline (1.1 equiv.), DMAP (0.5 equiv.), DIPEA (2.0 equiv.), and stirred at reflux temperature until complete consumption of the acyl halide was evident. The solvent was evaporated under reduced pressure and the residue was taken up in EtOAc, washed with 1 M aqueous NaOH, H_2_O, 1 M aqueous HCl, brine, and the organic layer dried (Na_2_SO_4_). The solvent was concentrated in vacuo and the residue purified by FCC with an appropriate eluent as indicated.

*Methyl (rel-9S,10S,12R)-12-methyl-9,10-dihydro-9,10-ethanoanthracene-12-carboxylate [38]* (**5**)

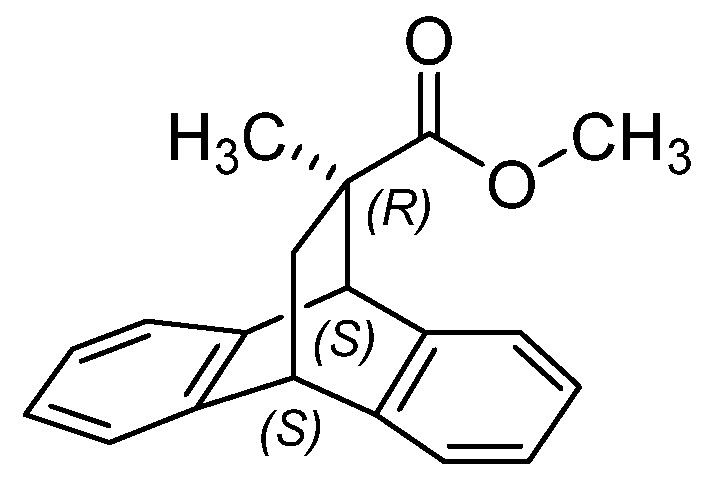



Methyl methacrylate (3.29 mL, 30.9 mmol, 1.1 eq.) was added to a suspension of AlCl_3_ (3.74 g, 28.1 mmol, 1.0 eq.) in dry DCM (100 mL), in a three-necked round-bottom flask under a positive pressure of N_2_. After 1 h of stirring, anthracene (5.00 g, 28.1 mmol, 1.0 eq.) was added portion-wise. The resulting mixture was stirred at room temperature for 72 h or until complete consumption of the anthracene was evident. The mixture was then poured over ice, the organic layer separated, washed with water (40 mL), dried (Na_2_SO_4_), and the solvent evaporated. The resulting residue was dissolved in DCM, absorbed onto silica gel and purified by FCC (eluent, 9:1 PE/Et_2_O) to afford the title compound as a transparent oil which solidified under high vacuum to give a white solid (5.67 g, 72%). LCMS (*m*/*z*): 301.1 [M + Na]^+^. System C HPLC: *t*_R_ 8.389 min, >95% purity (214 & 254 nm). ^1^H NMR (CDCl_3_) δ 7.32–7.28 (m, 1H), 7.26–7.22 (m, 2H), 7.19 (dd, *J* = 6.9, 1.6 Hz, 1H), 7.13–7.08 (m, 2H), 7.08–7.01 (m, 2H), 4.40 (s, 1H), 4.25 (t, *J* = 2.7 Hz, 1H), 3.53 (s, 3H) 2.71 (dd, *J* = 12.7, 3.0 Hz, 1H), 1.39 (dd, *J* = 12.7, 2.5 Hz, 1H), 1.05 (s, 3H). ^13^C NMR (CDCl_3_) δ 177.2, 143.8, 143.2, 141.6, 140.5, 126.4, 126.2, 126.2, 125.7, 125.6, 124.7, 123.6, 123.2, 52.9, 52.1, 44.5, 38.9, 26.7.

*rel-(9S,10S,12R)-12-Methyl-9,10-dihydro-9,10-ethanoanthracene-12-carboxylic acid* (**6**)

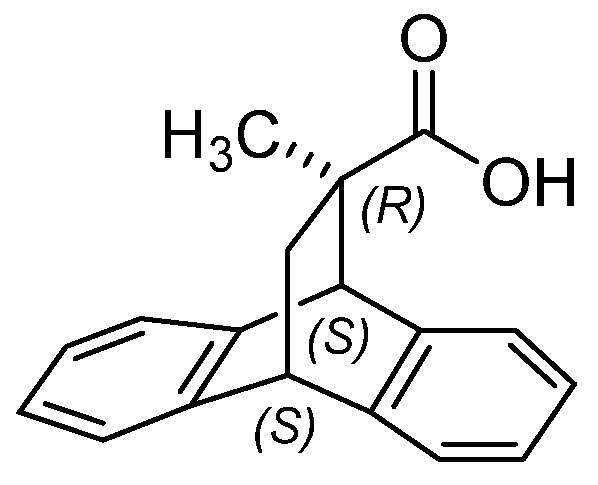



Synthesised according to general procedure C using LiOH.H_2_O (2.15 g, 89.8 mmol). After acidification, the precipitated solids were washed with water and recrystallised from EtOAc/PE, affording the compound as white needles (4.75 g, quantitative). LCMS (*m*/*z*): 263.1 [M − H]^+^. System C HPLC: *t*_R_ 7.181 min, >95% purity (214 & 254 nm). ^1^H NMR (CDCl_3_) δ 7.31–7.28 (m, 1H), 7.27–7.23 (m, 3H), 7.23–7.19 (m, 1H), 7.12 (ddd, *J* = 6.1, 5.6, 3.7 Hz, 2H), 7.08 (dd, *J* = 7.2, 1.5 Hz, 1H), 7.06–7.01 (m, 1H), 4.37 (s, 1H), 4.26 (t, *J* = 2.7 Hz, 1H), 2.62 (dd, *J* = 12.7, 3.0 Hz, 1H), 1.40 (dd, *J* = 12.7, 2.5 Hz, 1H), 1.07 (s, 3H). ^13^C NMR (CDCl_3_) δ 181.7, 143.6, 143.3, 141.3, 140.5, 126.5, 126.3, 126.2, 125.8, 125.7, 125.2, 123.5, 123.3, 52.6, 48.5, 44.5, 38.7, 26.9. Analytical data including ^1^H NMR and ^13^C NMR spectra are in accordance with those published [26,39].

*rel-(9R,10R,12S)-N-(2,6-Dichloro-3-methylphenyl)-12-methyl-9,10-dihydro-9,10-ethanoanthracene-12-carboxamide* (*rac*-**2**)

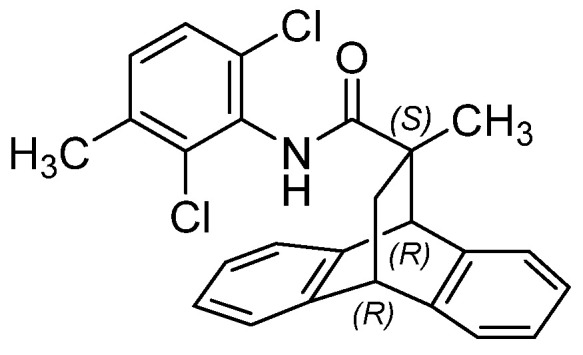



Synthesised according to general procedure D using oxalyl chloride (243 µL, 2.84 mmol), *rel*-(9*S*,10*S*,12*R*)-12-methyl-9,10-dihydro-9,10-ethanoanthracene-12-carboxylic acid (**6**) (375 mg, 1.42 mmol), DMAP (4.32 mg, 35.4 mmol), DIPEA (120 µL, 707 µmol) and 2,6-dichloro-3-methylaniline (65.4 mg, 371 µmol). Purification by FCC (eluent, 10:1 PE/EtOAc) afforded the racemic compound as a white solid (75 mg, 50%). LCMS (*m*/*z*): 446.1 [M + Na]^+^, 423.1 [M + H]^+^_._ System C HPLC: *t*_R_ 8.496 min, >95% purity (214 & 254 nm). HRMS (*m*/*z*): C_25_H_21_C_l2_NO: requires 422.1083 [M + H]^+^; found 422.1073. ^1^H NMR (CDCl_3_) δ 7.40–7.37 (m, 1H), 7.34–7.31 (m, 1H), 7.30–7.25 (m, 2H), 7.18–7.11 (m, 3H), 7.08–7.01 (m, 3H), 6.89 (s, 1H), 4.52 (s, 1H), 4.34 (t, *J* = 2.6 Hz, 1H), 2.64 (dd, *J* = 12.5, 2.8 Hz, 1H), 2.29 (s, 3H), 1.66 (dd, *J* = 12.4, 2.7 Hz, 1H), 1.18 (s, 3H). ^13^C NMR (CDCl_3_) δ 174.7, 143.3, 143.2, 141.5, 141.4, 136.1, 133.8, 132.2, 130.8, 129.6, 127.4, 126.5, 126.4, 126.2, 125.9, 125.9, 125.7, 123.5, 123.2, 52.7, 49.2, 44.7, 40.3, 28.3, 20.5. Analytical data including ^1^H NMR and ^13^C NMR spectra are in accordance with those published [26].

*(1R,2S,5R)-2-Isopropyl-5-methylcyclohexyl methacrylate* (**13**)

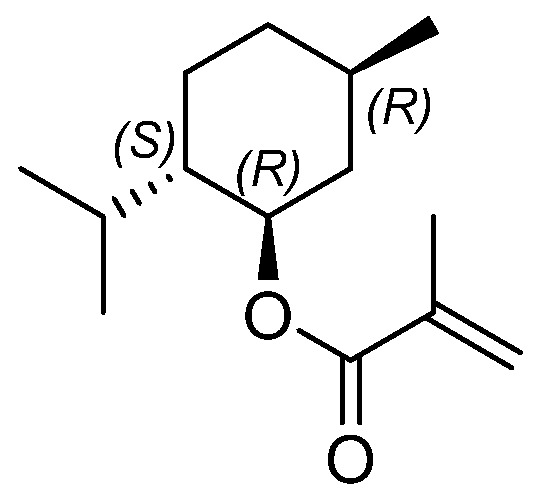



Synthesised according to general procedure A using methacrylic acid (1.62 mL, 19.2 mmol), (1*R*,2*S*,5*R*)-2-isopropyl-5-methylcyclohexan-1-ol (3.00 g, 19.2 mmol), EDC (4.05 g, 21.2 mmol) and DMAP (117 mg, 960 µmol). Purification by FCC (eluent, DCM) gave the title compound as a transparent oil (3.20 g, 74%). LCMS (*m*/*z*): 225.2 [M + H]^+^. System C HPLC: *t*_R_ 9.042 min, >95% purity (214 & 254 nm). ^1^H NMR (CDCl_3_) δ 6.23–6.20 (m, 1H), 5.81 (dd, *J* = 2.0, 1.2 Hz, 1H), 3.39 (td, *J* = 10.4, 4.3 Hz, 1H), 2.16 (dtd, *J* = 14.0, 7.0, 2.7 Hz, 1H), 1.99 (dt, *J* = 1.5, 0.9 Hz, 3H), 1.95–1.91 (m, 1H), 1.61 (ddd, *J* = 16.1, 14.6, 7.9 Hz, 2H), 1.45–1.37 (m, 2H), 1.14–1.05 (m, 1H), 0.96 (dd, *J* = 12.1, 4.1 Hz, 1H), 0.94–0.88 (m, 6H), 0.80 (dd, *J* = 7.0, 0.9 Hz, 3H). ^13^C NMR (CDCl_3_) δ 163.2, 135.9, 129.1, 71.7, 50.3, 45.2, 34.7, 31.8, 25.9, 23.3, 22.3, 21.1, 16.2. Analytical data in accordance with those published [54].

*(1R,2S,5R)-2-Isopropyl-5-methylcyclohexyl-12-methyl-9,10-dihydro-9,10-ethanoanthracene-12-carboxylate* (**14**)

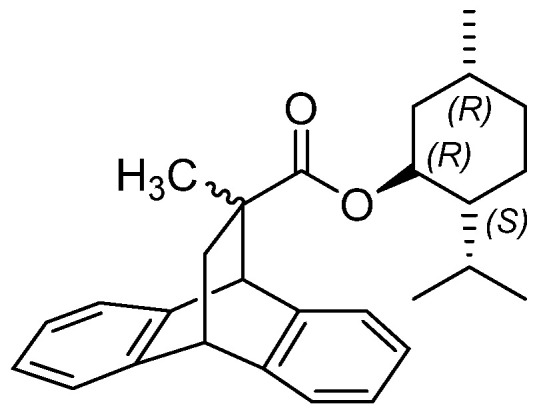



Synthesised according to general procedure B using TiCl_4_ (4.38 µL, 3.93 mmol), (1*R*,2*S*,5*R*)-2-isopropyl-5-methylcyclohexyl methacrylate (**13**) (440 mg, 1.96 mmol) and anthracene (350 mg, 1.96 mmol). Purification by FCC (eluent, 10:1 PE/EtOAc] gave the anthracene ester derivative as a light-yellow oil (400 mg, 51%, d.e. >70%). LCMS (*m*/*z*): 401.1 [M − H]^‒^. System C HPLC: *t*_R_ 2.204 min, >95% purity (214 & 254 nm). ^1^H NMR (CDCl_3_) δ 7.30–7.27 (m, 1H), 7.25–7.18 (m, 3H), 7.11–7.07 (m, 2H), 7.07–6.98 (m, 2H, H3), 4.48 (td, *J* = 10.8, 4.3 Hz, 1H), 4.38 (s, 1H), 4.23 (t, *J* = 2.6 Hz, 1H), 2.70 (dd, *J* = 12.7, 3.0 Hz, 1H), 1.86 (tt, *J* = 11.3, 3.5 Hz, 1H), 1.67–1.57 (m, 3H), 1.41–1.36 (m, 2H), 1.36–1.32 (m, 1H), 1.02 (s, 3H), 0.98 (dd, *J* = 13.1, 3.2 Hz, 1H), 0.91 (d, *J* = 7.0 Hz, 3H), 0.88–0.85 (m, 2H), 0.82 (d, *J* = 6.5 Hz, 3H), 0.68 (d, *J* = 6.9 Hz, 3H). ^13^C NMR (CDCl_3_) δ 176.3, 143.9, 143.4, 141.5, 141.0, 126.3, 126.1, 126.0, 125.5, 125.5, 125.2, 123.4, 123.2, 74.7, 52.9, 47.1, 44.6, 40.6, 38.8, 34.3, 31.4, 27.3, 26.0, 23.2, 22.1, 21.1, 16.1.

*(R)-N-(2-Hydroxy-1-phenylethyl)methacrylamide [55]* (**16**)

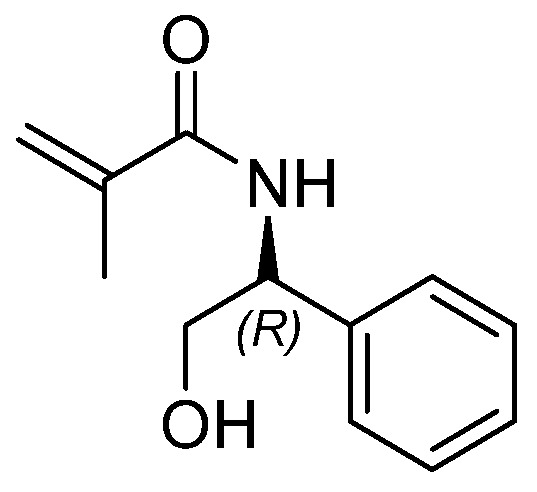



KO*t*-Bu (3.27 g, 29.2 mmol) was dissolved in THF (100 mL; technical grade, containing ca. 0.2% H_2_O) with stirring in air at rt for 1 min. Methyl methacrylate (1.55 mL, 14.6 mmol) and (*R*)-2-amino-2-phenylethan-1-ol (2.00 g, 14.6 mmol) were added immediately and the mixture was stirred at rt for 1 h. After evaporating the THF under reduced pressure, H_2_O (75 mL) and DCM (75 mL) were added and the organic layer was separated and dried (Na_2_SO_4_). The solvent was evaporated under reduced pressure and residue was purified by FCC (eluent, 1:10 DCM/MeOH) to give the corresponding amide as a yellow/orange solid (2.45 g, 89%). LCMS (*m*/*z*): 206.1 [M + H]^+^. System C HPLC: *t*_R_ 4.465 min, >95% purity (214 & 254 nm). ^1^H NMR (CDCl_3_) δ 7.38–7.33 (m, 2H), 7.32–7.28 (m, 3H), 6.57 (d, *J* = 5.6 Hz, 1H), 5.75 (s, 1H), 5.37–5.36 (m, 1H), 5.10 (dt, *J* = 7.0, 5.0 Hz, 1H), 3.89 (d, *J* = 4.9 Hz, 2H), 2.96 (s, 1H), 1.98–1.97 (m, 3H). ^13^C NMR (CDCl_3_) δ 168.9, 139.8, 139.2, 129.0, 128.0, 126.8, 120.3, 66.6, 56.1, 18.8. Analytical data in accordance with those published [55].

*N-((R)-2-Hydroxy-1-phenylethyl)-11-methyl-9,10-dihydro-9,10-ethanoanthracene-11-carboxamide* (**17**)

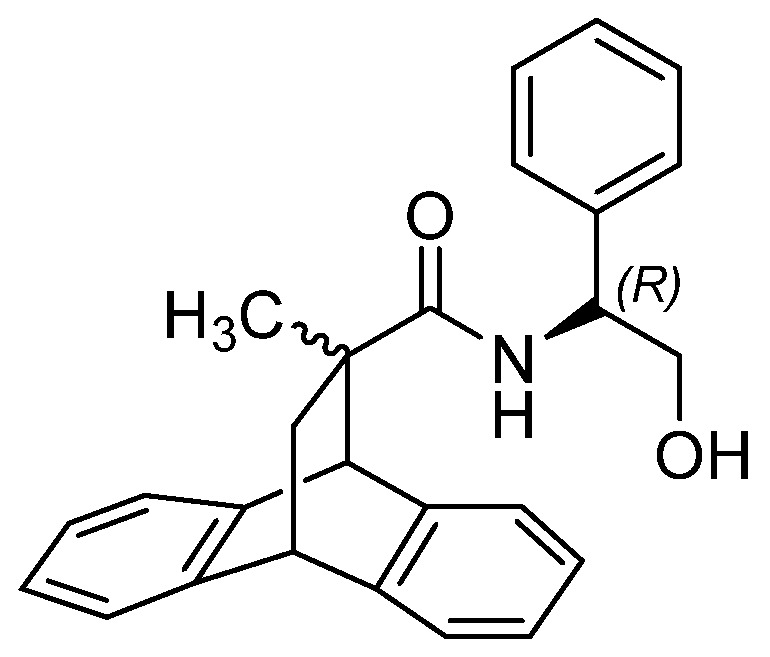



Synthesised according to general procedure B using TiCl_4_ (1.45 mL, 13.0 mmol), (*R*)-*N*-(2-hydroxy-1-phenylethyl)methacrylamide (1.34 g, 6.51 mmol) (**16**) and anthracene (1.16 g, 6.51 mmol). Residue chromatographed with 5:1 PE/EtOAc to afford a pair of diastereomers (2:1 ratio) as a transparent oil (2.00 g, 80%). LCMS (*m*/*z*): 384.2 [M + H]^+^. System C HPLC: *t*_R_ 7.254min, >95% purity (214 & 254 nm). ^1^H NMR (401 MHz, CDCl_3_) δ 7.36–7.25 (m, 12H), 7.20–7.11 (m, 6H), 7.08–6.99 (m, 4H), 6.04 (s, 0.5H), 5.75 (s, 1H), 4.86 (dt, *J* = 12.5, 5.8 Hz, 1.7H), 4.34 (d, *J* = 2.2 Hz, 3H), 3.80 (dd, *J* = 4.6, 2.4 Hz, 1H), 3.68 (dd, *J* = 11.3, 5.6 Hz, 1H), 3.59 (dd, *J* = 11.3, 3.9 Hz, 1H), 2.58 (dd, *J* = 12.8, 3.0 Hz, 0.7H), 2.38 (dd, *J* = 13.1, 3.0 Hz, 1H), 1.59 (dd, *J* = 13.2, 2.5 Hz, 1H), 1.55 (dd, *J* = 13.4, 3.0 Hz, 0.6H), 1.10 (s, *J* = 2.6 Hz, 1.4H), 1.09 (s, 3H).

*(R)-4,4-Dimethyl-2-oxotetrahydrofuran-3-yl methacrylate* ((*R*)-**19**)

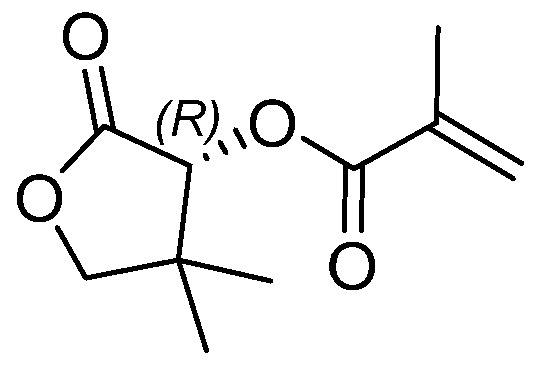



Synthesised according to general procedure A using methacrylic acid (1.49 g, 17.3 mmol), (*R*)-3-hydroxy-4,4-dimethyldihydrofuran-2(3*H*)-one (2.25 g, 17.3 mmol), EDC (3.31 g, 17.3 mmol) and DMAP (106 mg, 864 µmol). Purification by FCC (eluent, DCM) gave the title compound as a transparent oil (3.55 g, 67%). LCMS (*m*/*z*): 221.1 [M + Na]^+^. System C HPLC: *t*_R_ 6.010 min, >95% purity (214 & 254 nm). ^1^H NMR (CDCl_3_) δ 6.26–6.15 (m, 1H), 5.68 (dd, *J* = 2.6, 1.2 Hz, 1H), 5.41 (d, *J* = 0.8 Hz, 1H), 4.09–4.01 (m, 2H), 1.98 (dd, *J* = 1.7, 0.8 Hz, 3H), 1.21 (s, 3H), 1.13 (s, 3H). ^13^C NMR (CDCl_3_) δ 172.4, 165.9, 135.1, 127.5, 76.2, 75.2, 23.1, 19.9, 18.2.

*(S)-4,4-Dimethyl-2-oxotetrahydrofuran-3-yl methacrylate* ((*S*)-**19**)

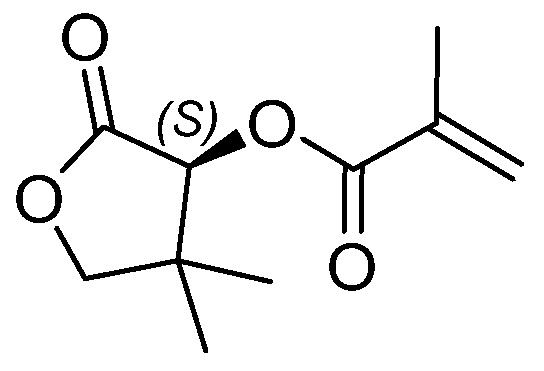



Synthesised according to general procedure A using methacrylic acid (2.32 g, 26.9 mmol), (*S*)-3-hydroxy-4,4-dimethyldihydrofuran-2(3*H*)-one (3.50 g, 26.9 mmol), EDC (5.16 g, 26.9 mmol), and DMAP (164 mg, 1.34 mmol). Purification by FCC (eluent, DCM) gave the title compound as a transparent oil (1.05 g, 69%). LCMS (*m*/*z*): 221.1 [M + Na]^+^. System C HPLC: *t*_R_ 6.010 min, >95% purity (214 & 254 nm). ^1^H NMR (CDCl_3_) δ 6.26–6.15 (m, 1H), 5.68 (dd, *J* = 2.6, 1.2 Hz, 1H), 5.41 (d, *J* = 0.8 Hz, 1H), 4.09–4.01 (m, 2H), 1.98 (dd, *J* = 1.7, 0.8 Hz, 3H), 1.21 (s, 3H), 1.13 (s, 3H). ^13^C NMR (CDCl_3_) δ 172.4, 165.9, 135.1, 127.5, 76.2, 75.2, 23.1, 19.9, 18.2.

*(R)-4,4-Dimethyl-2-oxotetrahydrofuran-3-yl-(S)-12-methyl-9,10-dihydro-9,10-ethanoanthracene-12-carboxylate* ((*S*)-**20**)

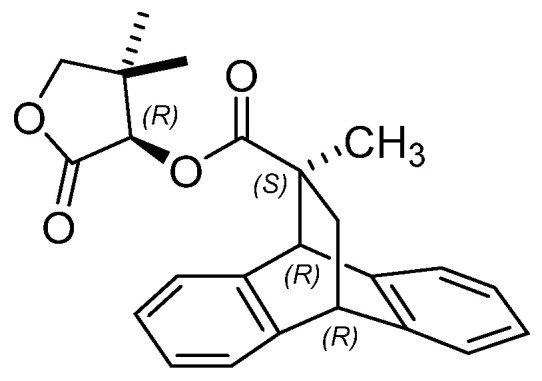



Synthesised according to general procedure B using TiCl_4_ (2.39 mL, 21.8 mmol), (*R*)-4,4-dimethyl-2-oxotetrahydrofuran-3-yl methacrylate ((*R*)-**19**) (2.06 g, 10.4 mmol) and anthracene (1.85 g, 10.4 mmol). Purification by FCC (eluent, DCM) afforded the title compound as a brown solid. Recrystallization from EtOH gave white crystals (3.25 g, 83%, d.e. ~90%). LCMS (*m*/*z*): 399.1 [M+Na]^+^. System C HPLC: *t*_R_ 8.320 min, >95% purity (214 & 254 nm). ^1^H NMR (401 MHz, CDCl_3_) δ 7.33 (dt, *J* = 5.4, 3.3 Hz, 1H), 7.30–7.26 (m, 2H), 7.24–7.21 (m, 1H), 7.16–7.10 (m, 2H), 7.10–7.02 (m, 2H), 5.18 (s, 1H), 4.40 (s, 1H), 4.30 (t, *J* = 2.7 Hz, 1H), 4.04 (d, *J* = 9.0 Hz, 1H), 3.97 (d, *J* = 9.0 Hz, 1H), 2.76 (dd, *J* = 12.8, 3.0 Hz, 1H), 1.50 (dd, *J* = 12.8, 2.6 Hz, 1H), 1.25 (s, 3H), 1.18 (s, 3H), 1.17 (s, 3H). ^13^C NMR (CDCl_3_) δ 175.6, 172.1, 143.7, 143.5, 141.2, 140.4, 126.6, 126.6, 125.7, 125.5, 124.8, 123.9, 123.4, 76.2, 75.5, 52.6, 49.0, 44.4, 39.2, 27.2, 23.0, 20.3.

*(S)-4,4-Dimethyl-2-oxotetrahydrofuran-3-yl-(R)-12-methyl-9,10-dihydro-9,10-ethanoanthracene-12-carboxylate* ((*R*)-**20**)

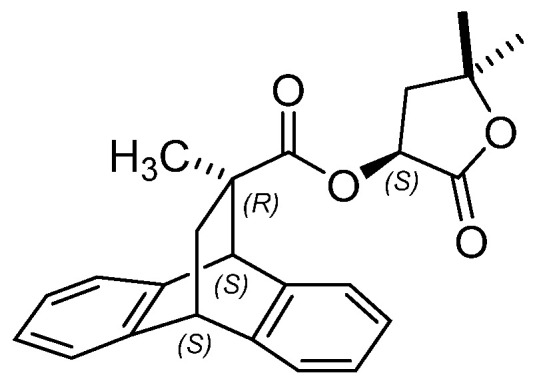



Synthesised according to general procedure B using TiCl_4_ (1.61 mL, 14.7 mmol), (*S*)-4,4-dimethyl-2-oxotetrahydrofuran-3-yl methacrylate ((*S*)-**19**) (1.39 g, 7.01 mmol) and anthracene (1.25 g, 7.01 mmol). Purification by FCC (eluent, DCM) afforded the title compound as a beige solid (2.15 g, 82%, d.e. ~83%) LCMS (*m*/*z*): 399.1 [M + Na]^+^. System C HPLC: *t*_R_ 8.320 min, >95% purity (214 & 254 nm). ^1^H NMR (CDCl_3_) δ 7.33 (dt, *J* = 5.4, 3.3 Hz, 1H), 7.30–7.26 (m, 2H), 7.24–7.21 (m, 1H), 7.16–7.10 (m, 2H), 7.10–7.02 (m, 2H), 5.18 (s, 1H), 4.40 (s, 1H), 4.30 (t, *J* = 2.7 Hz, 1H), 4.04 (d, *J* = 9.0 Hz, 1H), 3.97 (d, *J* = 9.0 Hz, 1H), 2.76 (dd, *J* = 12.8, 3.0 Hz, 1H), 1.50 (dd, *J* = 12.8, 2.6 Hz, 1H), 1.25 (s, 3H), 1.18 (s, 3H), 1.17 (s, 3H). ^13^C NMR (CDCl_3_) δ 175.6, 172.1, 143.7, 143.5, 141.2, 140.4, 126.6, 126.6, 125.7, 125.5, 124.8, 123.9, 123.4, 76.2, 75.5, 52.6, 49.0, 44.4, 39.2, 27.2, 23.0, 20.3.

*(S)-12-Methyl-9,10-dihydro-9,10-ethanoanthracene-12-carboxylic acid* ((*S*)-**21**)

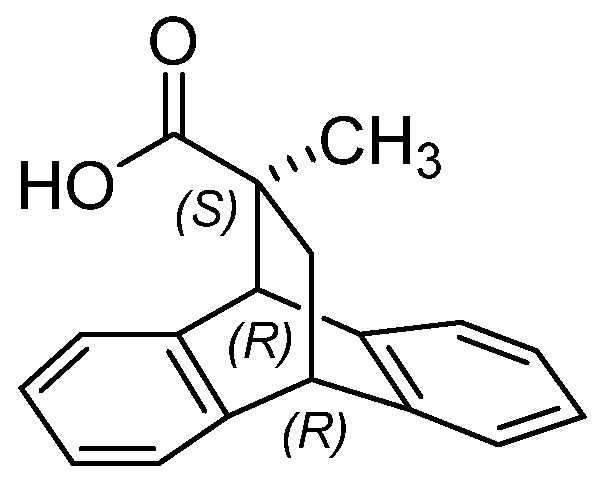



Synthesised according to general procedure C using (*R*)-4,4-Dimethyl-2-oxotetrahydrofuran-3-yl-(*S*)-12-methyl-9,10-dihydro-9,10-ethanoanthracene-12-carboxylate ((*S*)-**20**) (3.00 g, 7.97 mmol) and NaOH (956 mg, 23.9 mmol). The aqueous phase was extracted with DCM, and the organic extracts were dried (Na_2_SO_4_) and evaporated to dryness to afford the desired acid as a white foam (2.05 g, 95%, e.e. ~88%). [α]_D_^25^ = −24.9° (*c* 1.0, CHCl_3_), ^lit^[α]_D_^20^ = −26.7° (*c* 1.08, CHCl_3_) [39], LCMS (*m*/*z*): 263.1 [M − H]^‒^. System C HPLC: *t*_R_ 7.181 min, >95% purity (214 & 254 nm). Analytical data including ^1^H NMR and ^13^C NMR spectra are in accordance with those reported by Camps et al. [39]

*(R)-12-Methyl-9,10-dihydro-9,10-ethanoanthracene-12-carboxylic acid* ((*R*)-**21**)

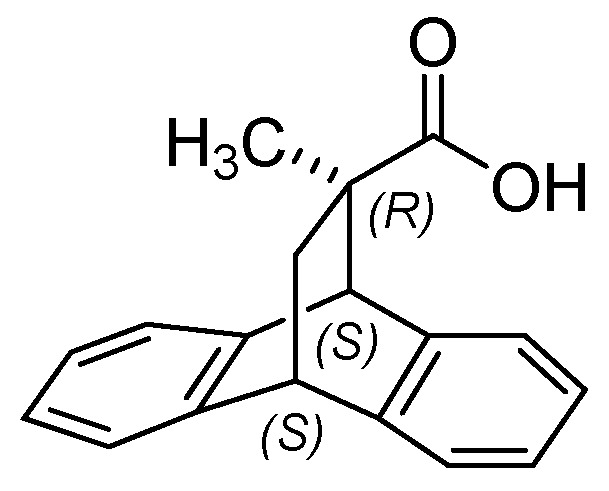



Synthesised according to general procedure C using (*S*)-4,4-Dimethyl-2-oxotetrahydrofuran-3-yl-(*S*)-12-methyl-9,10-dihydro-9,10-ethanoanthracene-12-carboxylate ((*R*)-**20**) (1.40 g, 3.72 mmol) and NaOH (446 mg, 11.2 mmol). The aqueous phase was extracted with DCM, and the organic extracts were dried (Na_2_SO_4_) and evaporated to dryness to afford the desired acid as a white solid (956 mg, 98%, e.e. ~83%). [α]_D_^25^ = +24.1° (*c* 1.0, CHCl_3_), LCMS (*m*/*z*): 263.1 [M − H]^‒^. System C HPLC: *t*_R_ 7.181 min, >95% purity (214 & 254 nm). ^1^H NMR (CDCl_3_) δ 7.31–7.28 (m, 1H), 7.27–7.23 (m, 3H), 7.23–7.19 (m, 1H), 7.12 (ddd, *J* = 6.1, 5.6, 3.7 Hz, 2H), 7.08 (dd, *J* = 7.2, 1.5 Hz, 1H) 7.06–7.01 (m, 1H), 4.37 (s, 1H), 4.26 (t, *J* = 2.7 Hz, 1H), 2.62 (dd, *J* = 12.7, 3.0 Hz, 1H), 1.40 (dd, *J* = 12.7, 2.5 Hz, 1H), 1.07 (s, 3H). ^13^C NMR (CDCl_3_) δ 181.7, 143.6, 143.3, 141.3, 140.5, 126.5, 126.3, 126.2, 125.8, 125.7, 125.2, 123.5, 123.3, 52.6, 48.5, 44.5, 38.7, 26.9.

*(9S,10R,12R)-N-(2,6-Dichloro-3-methylphenyl)-12-methyl-9,10-dihydro-9,10-ethanoanthracene-12-carboxamide* ((*S*)-**2**)

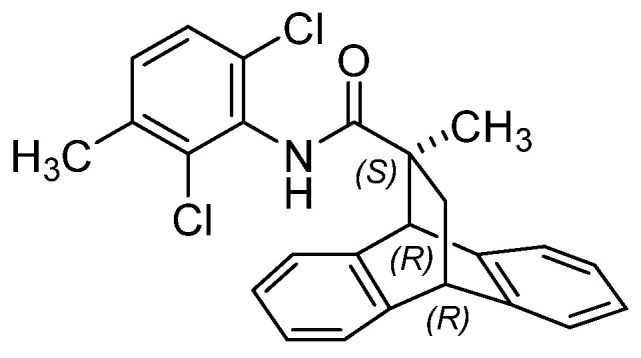



Synthesised according to general procedure D using (*S*)-12-methyl-9,10-dihydro-9,10-ethanoanthracene-11-carbonyl chloride ((*S*)-**21**) (260 mg, 919 µmol), 2,6-dichloro-3-methylaniline (162 mg, 920 µmol), and DMAP (56.2 mg, 460 µmol). Purification by FCC (eluent, 1:10 EtOAc/PE) afforded the title compound as a white solid (75 mg, 19%, e.e. 90%). LCMS (*m*/*z*): 446.1 [M + Na]^+^, 423.1 [M + H]^+^_._ System C HPLC: *t*_R_ 8.496 min, >95% purity (214 & 254 nm). HRMS (*m*/*z*): C_25_H_21_C_l2_NO: requires 422.1083 [M + H]^+^; found 422.1073. ^1^H NMR (CDCl_3_) δ 7.40–7.37 (m, 1H), 7.34–7.31 (m, 1H), 7.30–7.25 (m, 2H), 7.18–7.11 (m, 3H), 7.08–7.01 (m, 3H), 6.89 (s, 1H), 4.52 (s, 1H), 4.34 (t, *J* = 2.6 Hz, 1H), 2.64 (dd, *J* = 12.5, 2.8 Hz, 1H), 2.29 (s, 3H), 1.66 (dd, *J* = 12.4, 2.7 Hz, 1H), 1.18 (s, 3H). ^13^C NMR (CDCl_3_) δ 174.7, 143.3, 143.2, 141.5, 141.4, 136.1, 133.8, 132.2, 130.8, 129.6, 127.4, 126.5, 126.4, 126.2, 125.9, 125.9, 125.7, 123.5, 123.2, 52.7, 49.2, 44.7, 40.3, 28.3, 20.5.

*(9R,10S,12S)-N-(2,6-Dichloro-3-methylphenyl)-12-methyl-9,10-dihydro-9,10-ethanoanthracene-12-carboxamide* ((*R*)-**2**)

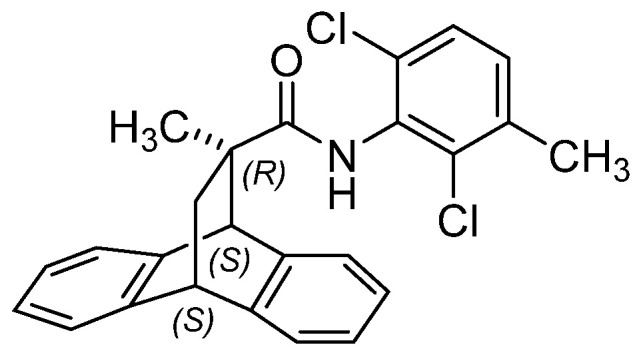



Synthesised according to general procedure D using (*R*)-12-methyl-9,10-dihydro-9,10-ethanoanthracene-11-carbonyl chloride ((*R*)-**21**) (135 mg, 477 µmol), 2,6-dichloro-3-methylaniline (84. 1 mg, 477 µmol), and DMAP (29.1 mg, 239 µmol). Purification by FCC (eluent, 1:10 EtOAc/PE) afforded the title compound as a white solid (50 mg, 25%, e.e. 84%). LCMS (*m*/*z*): 446.1 [M + Na]^+^, 423.1 [M + H]^+^_._ System C HPLC: *t*_R_ 8.496 min, >95% purity (214 & 254 nm). HRMS (*m*/*z*): C_25_H_21_C_l2_NO: requires 422.1083 [M + H]^+^; found 422.1073. ^1^H NMR (CDCl_3_) δ 7.40–7.37 (m, 1H), 7.34–7.31 (m, 1H), 7.30–7.25 (m, 2H), 7.18–7.11 (m, 3H), 7.08–7.01 (m, 3H), 6.89 (s, 1H), 4.52 (s, 1H), 4.34 (t, *J* = 2.6 Hz, 1H), 2.64 (dd, *J* = 12.5, 2.8 Hz, 1H), 2.29 (s, 3H), 1.66 (dd, *J* = 12.4, 2.7 Hz, 1H), 1.18 (s, 3H). ^13^C NMR (CDCl_3_) δ 174.7, 143.3, 143.2, 141.5, 141.4, 136.1, 133.8, 132.2, 130.8, 129.6, 127.4, 126.5, 126.4, 126.2, 125.9, 125.9, 125.7, 123.5, 123.2, 52.7, 49.2, 44.7, 40.3, 28.3, 20.5.

### 3.3. Pharmacological Characterisation

#### 3.3.1. Materials

Dulbecco’s modified Eagle’s medium, Flp-In CHO cells, and hygromycin B (Invitrogen, Carlsbad, CA, USA). Fetal bovine serum (FBS) (ThermoTrace, Melbourne, VIC, Australia). All other reagents (Sigma Aldrich, St. Louis, MO, USA).

#### 3.3.2. General Cell Culture

FlpIn Chinese Hamster Ovary (CHO) cells (Invitrogen, Carlsbad, CA, USA) were grown in DMEM supplemented with 10% FBS and maintained at 37 °C in a humidified incubator (5% CO_2_). The Flp-In-CHO cells were transfected with the pOG44 vector encoding Flp recombinase and the pDEST vector encoding the D_2L_R or the hD_1_R at a ratio of 9:1 using PEI as transfection reagent. Following transfection (24 h), cells were subcultured and the medium supplemented with 700 mg/mL HygroGold (Invivogen) as selection agent, to obtain cells stably expressing the D_1_R or D_2L_R. FlpIn Chinese Hamster Ovary CHO cells stably expressing the hD_1_R or hD_2L_R were grown and maintained in Dulbecco’s modified Eagle’s medium (DMEM) supplemented with 10% FBS, and 200 μg/mL of Hygromycin-B, and maintained at 37 °C in a humidified incubator (5% CO_2_).

##### Cell Culture and Transfection for cAMP Assay

FlpIn CHO cells stably expressing the hD_1_R were maintained in DMEM supplemented with 5% fetal calf serum and 0.2 mg/mL hygromycin at 37 °C in a humidified incubator (5% CO_2_). For transfection, the cells were grown in 10 cm culture dishes until 60% confluent. A mixture of 4 μg plasmid DNA containing BRET-based cAMP (CAMYEL) biosensor construct [52] and 25 μg 20 kDa linear PEI in 500 μL 150 mM NaCl was added into a dish of cells.

#### 3.3.3. cAMP Assay Measurement

Cellular cAMP levels were measured with the CAMYEL BRET-based biosensor for cAMP [52]. One day following transfection, cells were trypsinised and seeded in white 96-well microplates, cultured for an additional day, rinsed (×2) with Hank’s Balanced Salt Solution (HBSS), then incubated in fresh HBSS. For the D_1_R functional assay, the cells were stimulated with DA together with addition of the modulators. The BRET signals were measured using a BMG Lumistar counter 30 min after stimulation. For the D_2_R functional assay, cells were stimulated with DA in the presence of 10 μM forskolin (final concentration). DA and modulators were added 15 min prior to stimulation and BRET signals were measured using a BMG Lumistar counter 30 min after stimulation. The BRET signal (BRET ratio) was detected at 445–505 nm and 505–565 nm using a LUMIstar Omega instrument (BMG LabTech, Offenburg, Germany), and quantified by calculating the ratio of light emitted at 535 ± 30 nm (YFP) to light emitted at 475 ± 30 nm (RLuc).

#### 3.3.4. Data Analysis of Allsotery

Simulations, statistical analyses and computerized nonlinear regression were performed using Prism 6.0 (GraphPad Prism 6.0b Software, San Diego, CA, USA).

Analysis of functional data. All concentration-response data were fitted to the following modified four-parameter Hill equation in order to derive potency estimates [56].

(1) $$E=Basal+\frac{\left(E_{max}-Basal\right)\cdot {\left[A\right]}^{nH}}{\left[A^{nH}\right]+EC_{50}^{nH}}$$

where *E* = the effect of the system; *nH* = Hill slope; *EC*_50_ = [*A*] (concentration of agonist) that provides the midpoint response between basal and maximal effect of DA or other agonists (*E_max_*), which are the lower and upper asymptotes of the response, respectively.

To determine the mode of interaction of compounds **2**, and optical isomers of **2** at the D_1_R in relation to the agonist DA, all data were analyzed using a complete operational model of allosterism and agonism according to Equation (2) [34]

(2) $$E=\frac{E_{m}(\tau_{A}\left[A\right]{\left(K_{B\;}+\alpha \beta \left[B\right])+\tau_{B}\left[B\right]K_{\;A}\right)}^{nH}}{{\left(\left[A\right]K_{B}+K_{A}K_{B}+K_{A}\left[B\right]+\alpha \left[A\right]\left[B\right]\right)}^{nH}+{\left(\tau_{A}\left[A\right]\left(K_{B}+\alpha \beta \left[B\right]\right)+\tau_{B}\left[B\right]K_{A}\right)}^{nH}}$$

where *E_m_* = maximum possible cellular response, [*A*] & [*B*] = concentrations of orthosteric and allosteric ligands, respectively, *K_A_* & *K_B_* = equilibrium dissociation constants of orthosteric and allosteric ligands, respectively, *τ_A_* & *τ_B_* = operational measures of orthosteric and allosteric ligand efficacy (which incorporate both signal efficiency and receptor density), respectively, *α* = binding cooperativity parameter between orthosteric and allosteric ligand, and *β* = magnitude of the allosteric effect of modulator on efficacy of orthosteric agonist.

The *K_A_* for DA was determined through receptor depletion by phenoxybenzamine alkylation as the proportional relationship of *R_T_* to measured *τ* whilst *K_A_* is invariant with receptor depletion. Thus, unique estimates of *K_A_* could be measured by direct fitting of the operational model to the family of concentration-response curve for DA [57,58]. A series of cAMP inhibition assays were conducted in cells which were pre-treated with phenoxybenzamine (an alkylating agent which is used to inhibit high affinity orthosteric interactions at the D_2_R) [59]. The data for DA evaluated in the presence of the alkylating agent were then fit to an operational model of receptor depletion in order to determine values of *K_A_* and *τ_A_* (log *K_A_* = −5.78 ± 0.16, log *τ_A_* = 1.84 ± 0.16). These data were used to constrain values of *K_A_* and *τ_A_* when we fitted the operational model of allostery (Equation (2)) to functional data. The value of the Hill slope (*nH*) was fixed to unity. The logarithms of affinity and cooperativity values are normally distributed, whereas the absolute values (antilogarithms) are not, and therefore all statistical analyses were performed on the logarithmic values. However, for ease of interpretation, allosteric parameter antilogarithms are highlighted in the main text [60].

## 4. Conclusions

In this study, we describe the first reported enantioenrichment of optical isomers of *rac*-**2** using chiral auxiliaries derived from enantiomers of 3-hydroxy-4,4-dimethyldihydrofuran-2(3*H*)-one and their pharmacological characterisation. Interestingly, (*R*)-**2** was shown to display 4-fold lower positive cooperativity with DA as compared to (*S*)-**2** and a 7-fold lower affinity for the D_1_R. Our findings illustrate the importance of further investigation into asymmetric syntheses of analogues of **2** and/or isolation of optically pure analogues as part of future SAR efforts aimed at developing enhanced D_1_R PAMs based on this scaffold.

## Figures and Tables

**Table 1:** Functional parameters for PAMs derived from cAMP BRET assay at the hD_1_R.

CPD	e.e. (%)	p*K_B_* (*K_B_,* µM) ^1^	Log*τ_B_* (*τ_B_*) ^2^	Log*αβ* (*αβ*) ^3^
*rac*-2	0	5.80 ± 0.10 (1.6)	0.32 ± 0.06 (2.1)	2.00 ± 0.12 (100)
(*S*)-2	90	5.99 ± 0.09 (1.0)	0.40 ± 0.06 (2.5)	2.10 ± 0.25 (125)
(*R*)-2	84	5.12 ± 0.06 (7.6) *^,^^	0.46 ± 0.08 (2.9)	1.51 ± 0.14 (31) *^,^^

^1^ Estimate of the negative logarithm of the equilibrium dissociation constant determined in an cAMP functional assay. ^2^ Estimate of the intrinsic efficacy of the modulator. ^3^ Estimate of the logarithm of the net cooperativity factor between the modulator and DA. Values represent mean ± S.D. from at least four independent experiments performed in duplicate. Significant differences parameter between (*R*)-**2** and *rac*-**2** * or (*S*)-**2** ^, *p* < 0.05, one-way ANOVA with Tukey’s Post-hoc test (GraphPad Prism Version 7).

## Data Availability

Data is contained within the article or Supplementary Materials.

## Supplementary Materials

The following are available online. Figure S1: Chiral HPLC chromatogram for Compound **5**, Figure S2: Chiral HPLC chromatogram for Compound (*S*)-**21**, Figure S3: Chiral HPLC chromatogram for Compound (*R*)-**21**, Figures S4–S7: HPLC & chiral HPLC chromatograms, ^1^H & ^13^C NMR spectra for 2, Figure S8: HPLC chromatogram (MeOH blank), Figures S9–S12: HPLC & chiral HPLC chromatograms, ^1^H & ^13^C NMR spectra for (*S*)-**2**, Figures S13–S16: HPLC & chiral HPLC chromatograms, ^1^H & ^13^C NMR spectra for (*R*)-**2**.

## Sample Availability

Samples of the compounds are available from the authors.
