# Supplementary material for: Enantioenriched Positive Allosteric Modulators Display Distinct Pharmacology at the Dopamine D1 Receptor
Source: Molecules. 2021 Jun 22;26(13):3799. doi: 10.3390/molecules26133799 (PMC8270344; doi:10.3390/molecules26133799)

Supplementary Material

Table of Contents

Figure S1 **Chiral HPLC chromatogram for Compound 5...….…..………..S2**

Figure S2 **Chiral HPLC chromatogram for Compound (*S*)-21…..………..S2**

Figure S3 **Chiral HPLC chromatogram for Compound (*R*)-21….………...S3**

Figures S4-S7 **HPLC & chiral HPLC chromatograms, ^{1}H & ^{13}C NMR spectra for 2..……………………………………..…………......S3-5**

Figure S8 **HPLC chromatogram (MeOH blank)………………………….....S5**

Figure S9-S12 **HPLC & chiral HPLC chromatograms, ^{1}H & ^{13}C NMR spectra for (*S*)-2……………………………………………….………......S6-7**

Figure S13-S16 **HPLC & chiral HPLC chromatograms, ^{1}H & ^{13}C NMR spectra for (*R*)-2...S8-9**

Figure S1. Chiral HPLC trace for Compound 5 (racemic carboxylic acid), obtained using 'Column A' as outlined in general information.

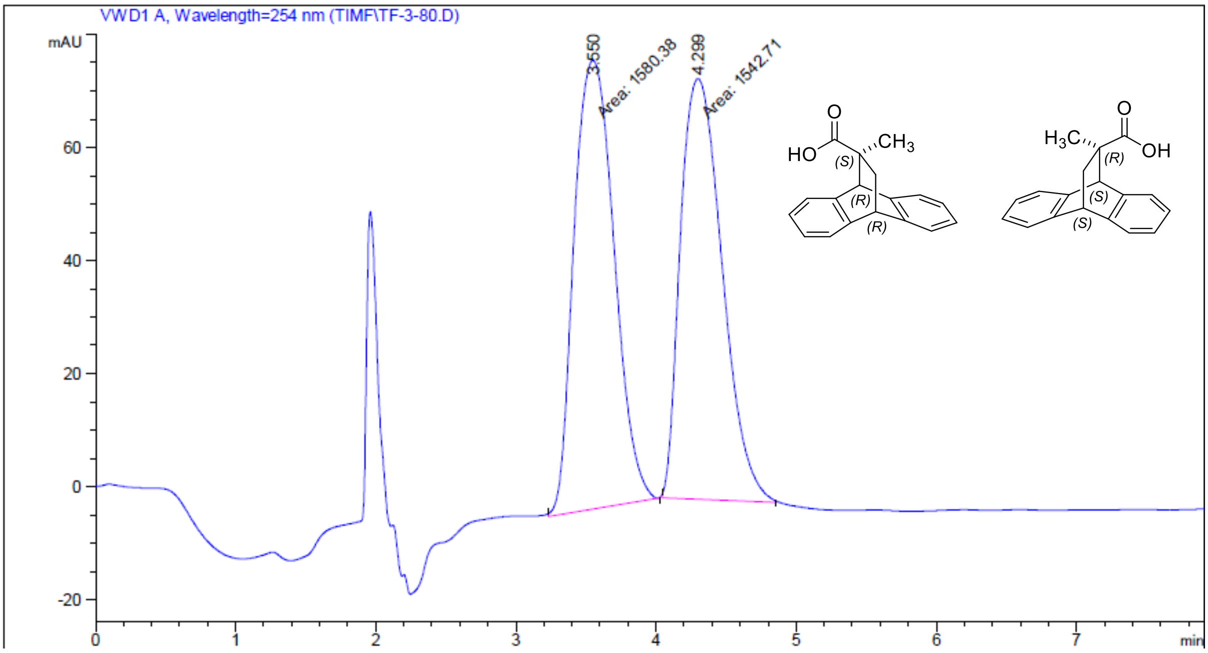

Figure S2. Chiral HPLC trace for Compound (*S*)-21 (carboxylic acid), obtained using 'Column A' as outlined in general information.

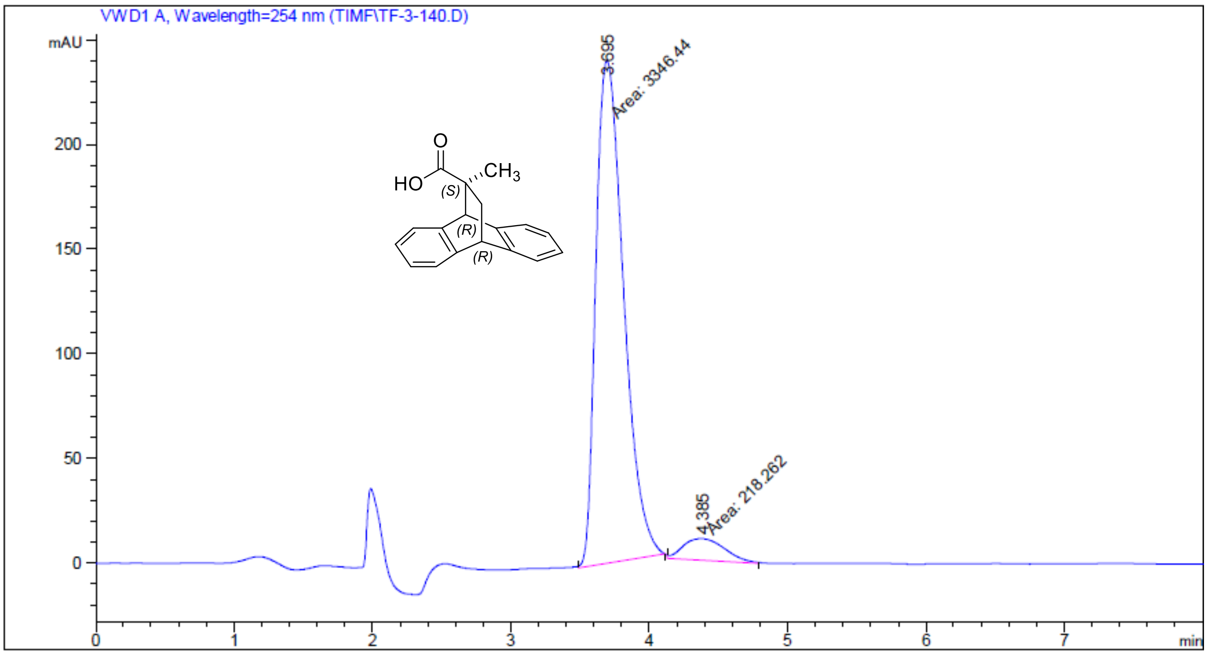

Figure S3. Chiral HPLC trace for Compound (*R*)-21 (carboxylic acid), obtained using 'Column A' as outlined in general information.

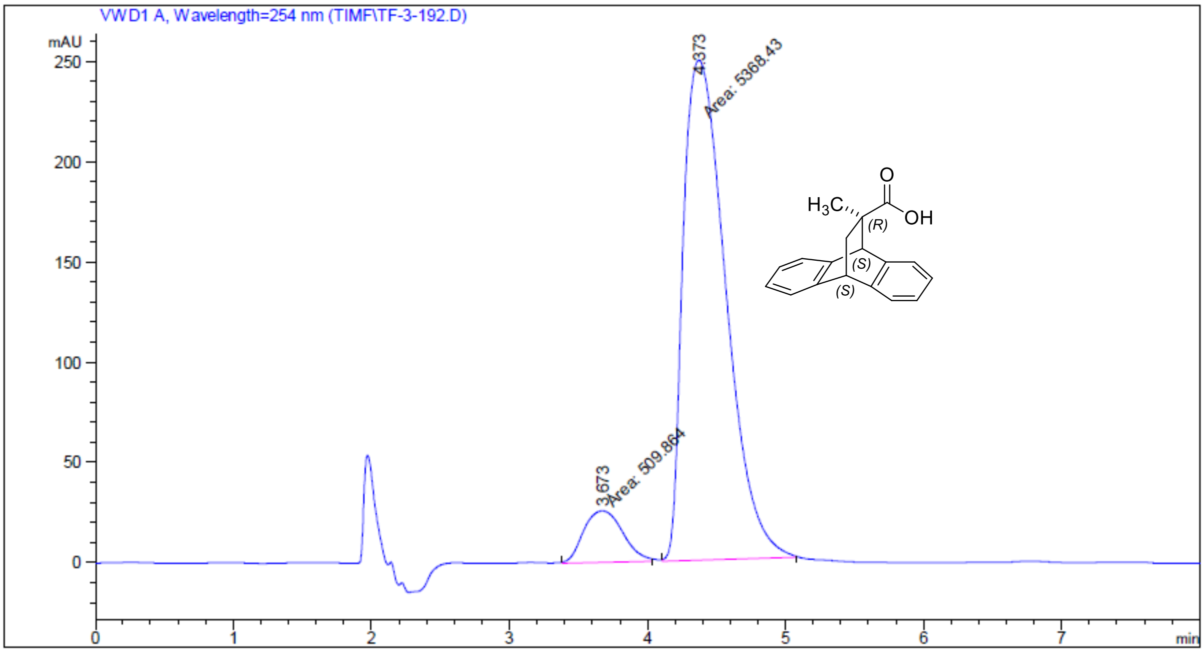

Figure S4. Analytical HPLC trace for Compound (*rac*-2).

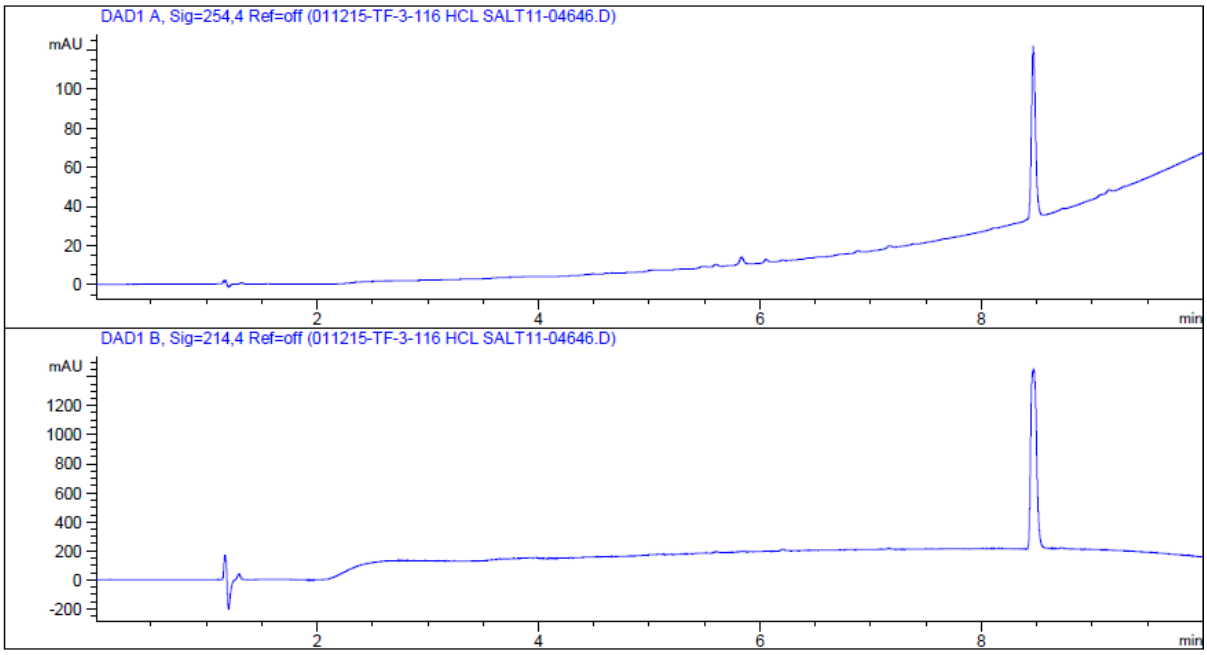

Figure S5. Chiral HPLC trace for Compound (*rac*-2), obtained using 'Column B' as outlined in general information.

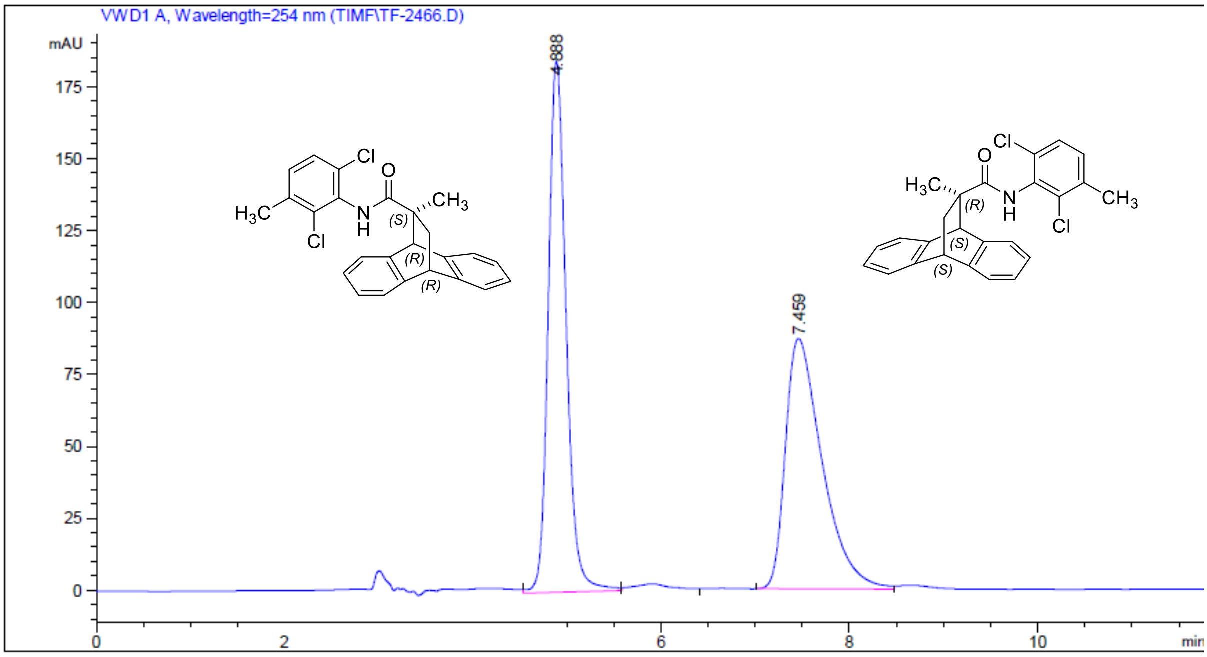

Figure S6. ^{1}H NMR spectrum for Compound (*rac*-2).

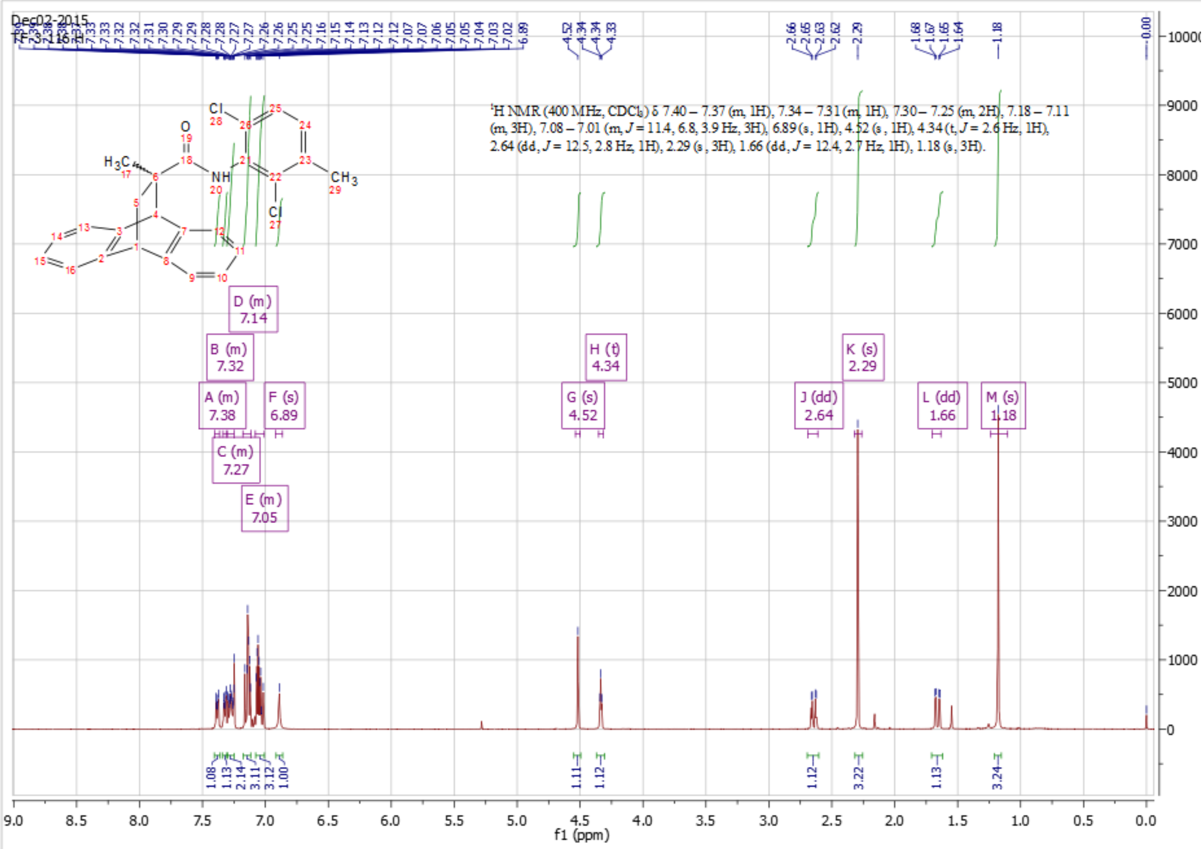

Figure S7. ^{13}C NMR spectrum for Compound (*rel*-2).

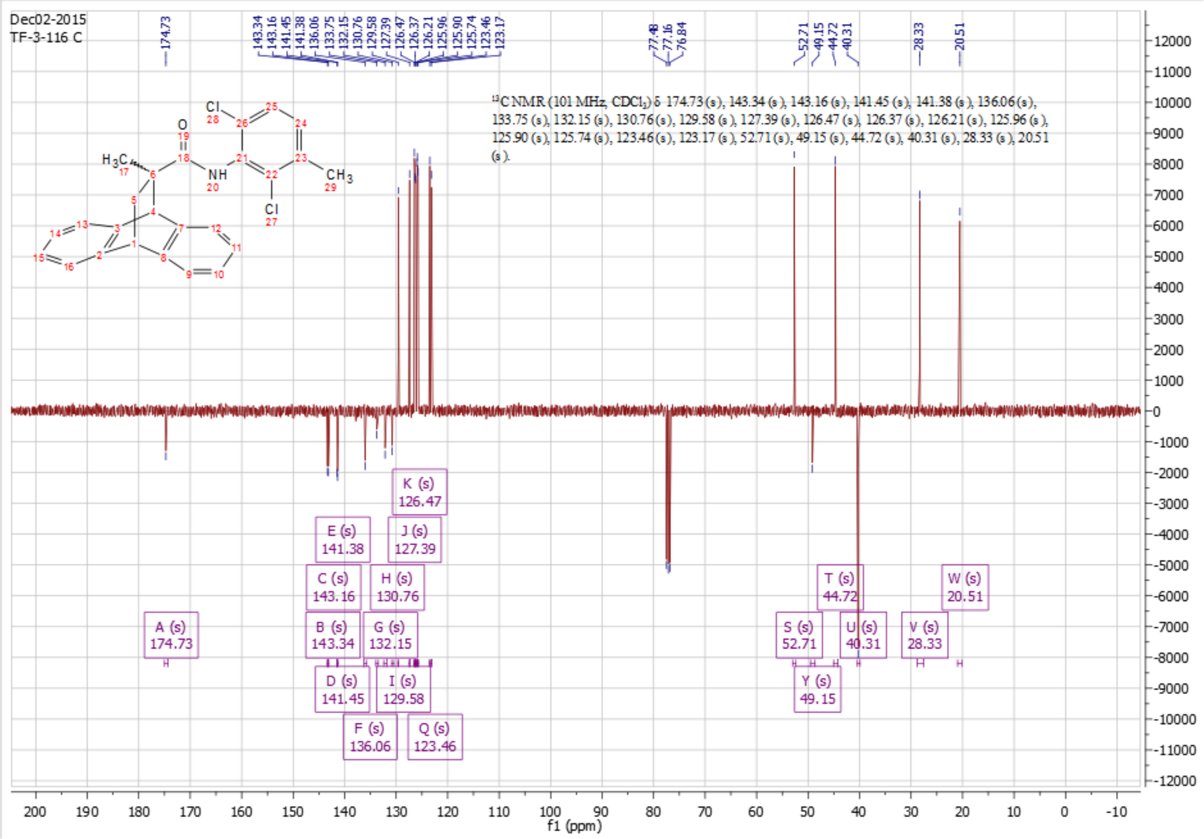

Figure S8 Analytical HPLC trace (MeOH BLANK).

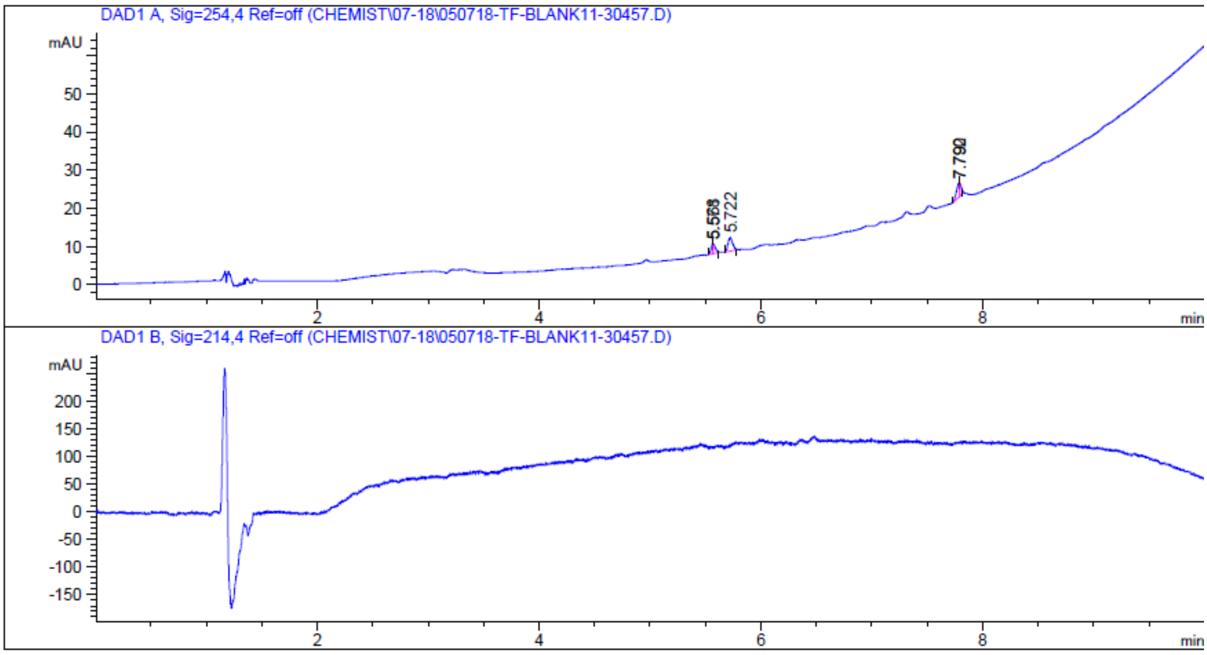

Figure S9. Analytical HPLC Trace for Compound (*S*)-2.

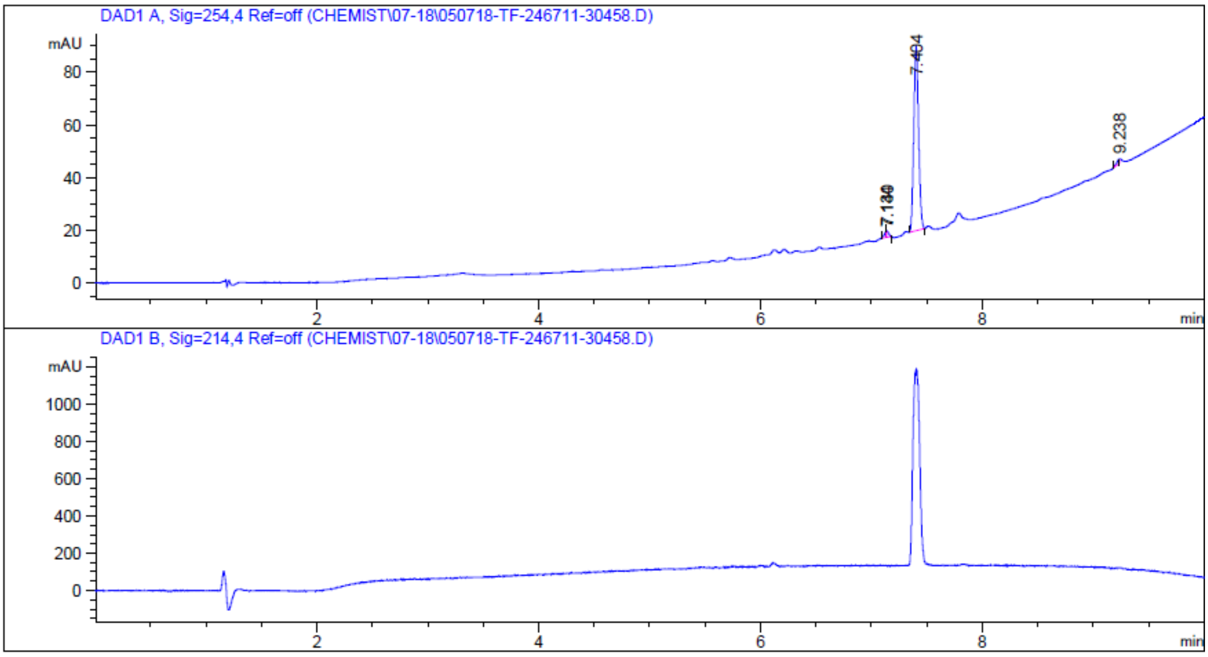

Figure S10. Chiral HPLC Trace for Compound (*S*)-2, obtained using 'Column B' as outlined in general information.

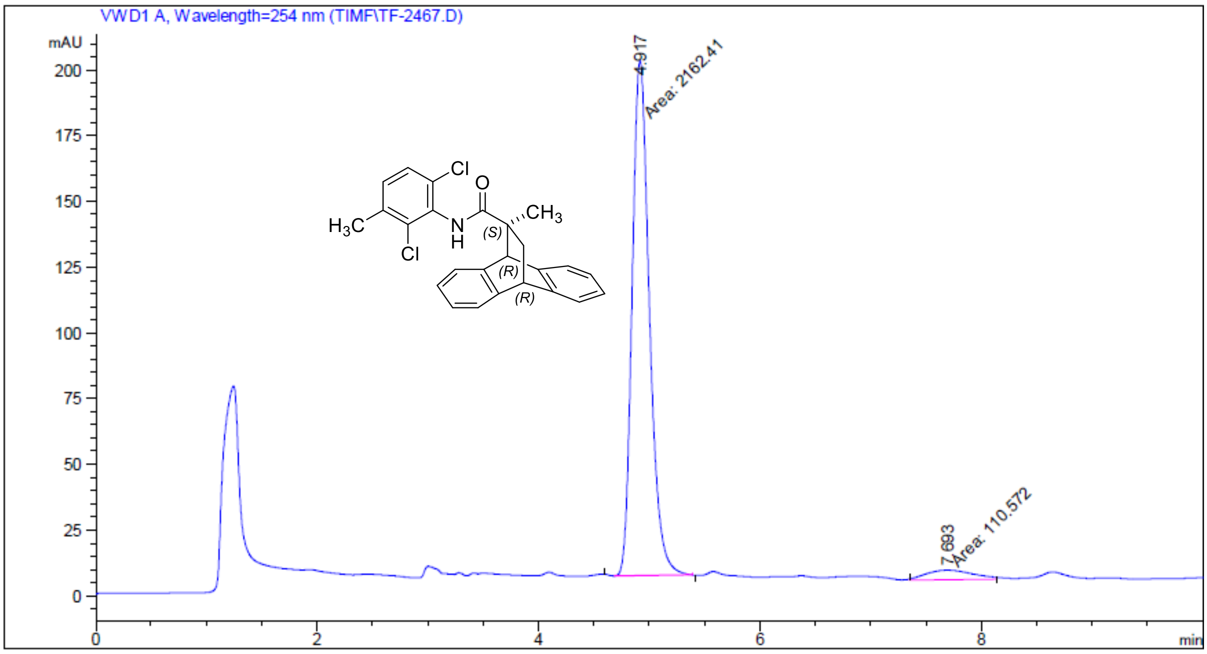

Figure S11. ^{1}H NMR spectrum for Compound (*S*)-2.

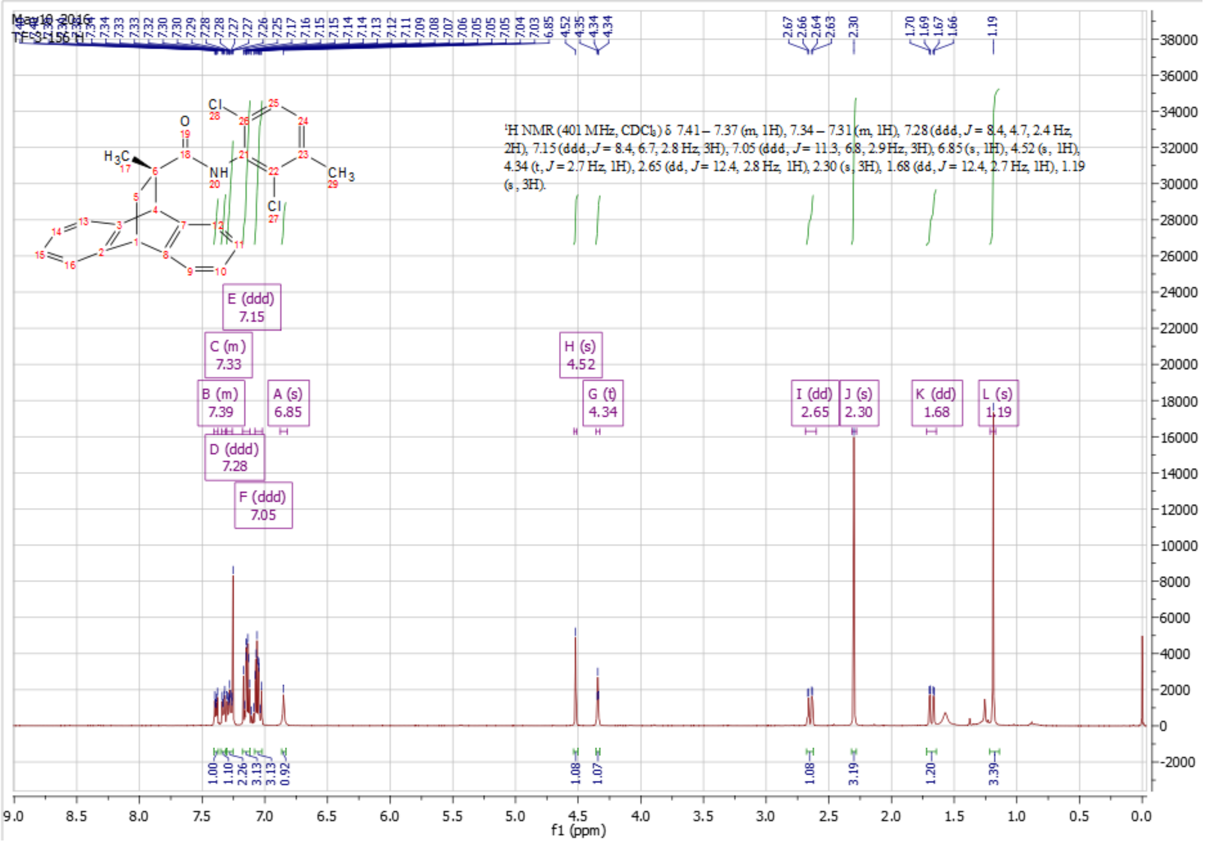

Figure S12. ^{13}C NMR spectrum for Compound (*S*)-2.

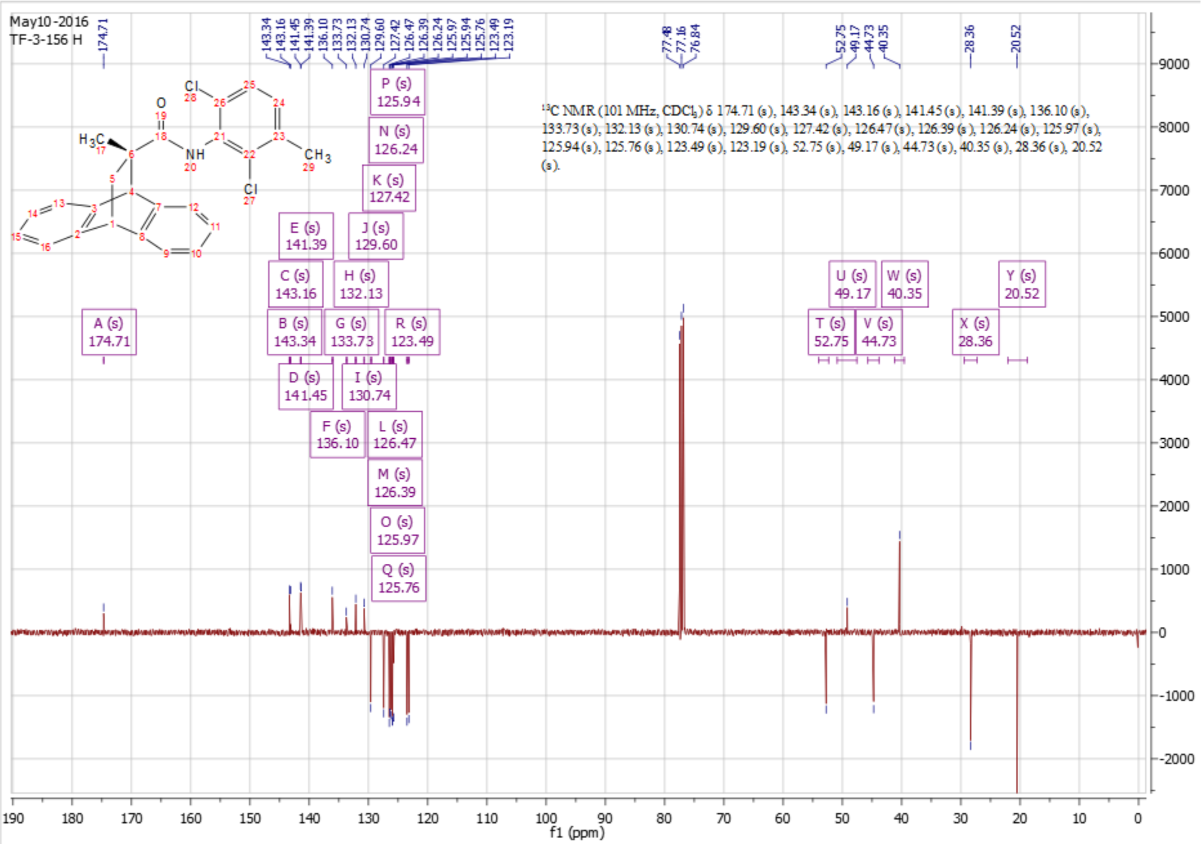

Figure S13. Analytical HPLC trace for Compound (*R*)-2.

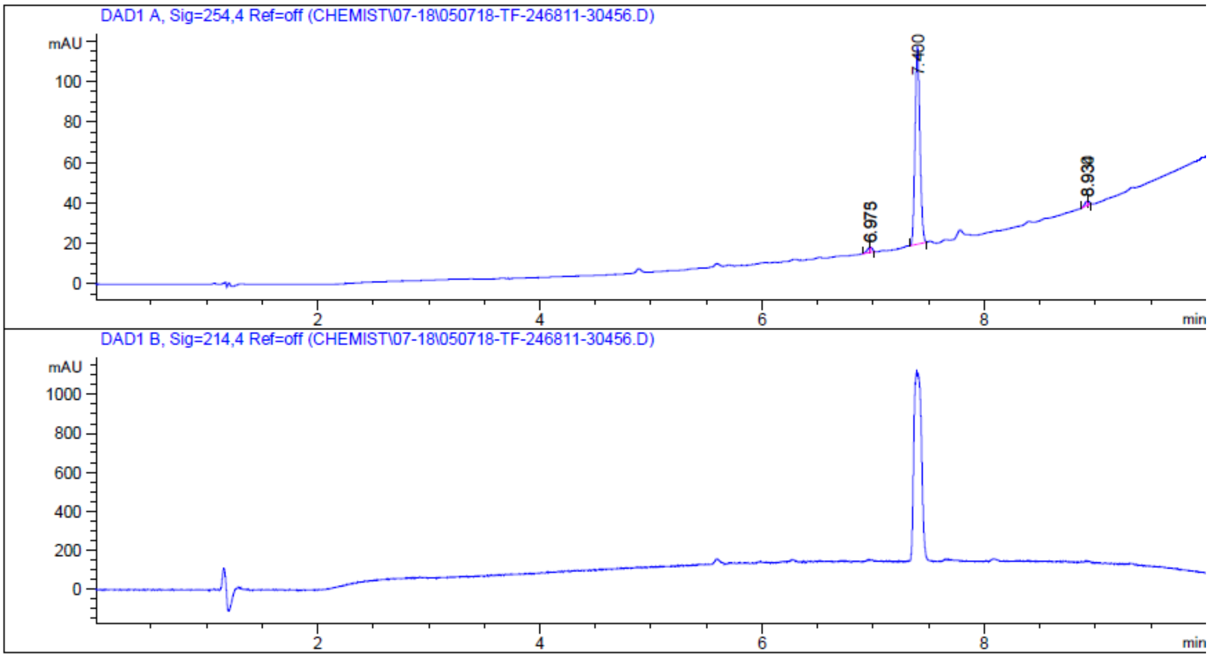

Figure S14. Chiral HPLC trace for Compound (*R*)-2, obtained using 'Column B' as outlined in general information.

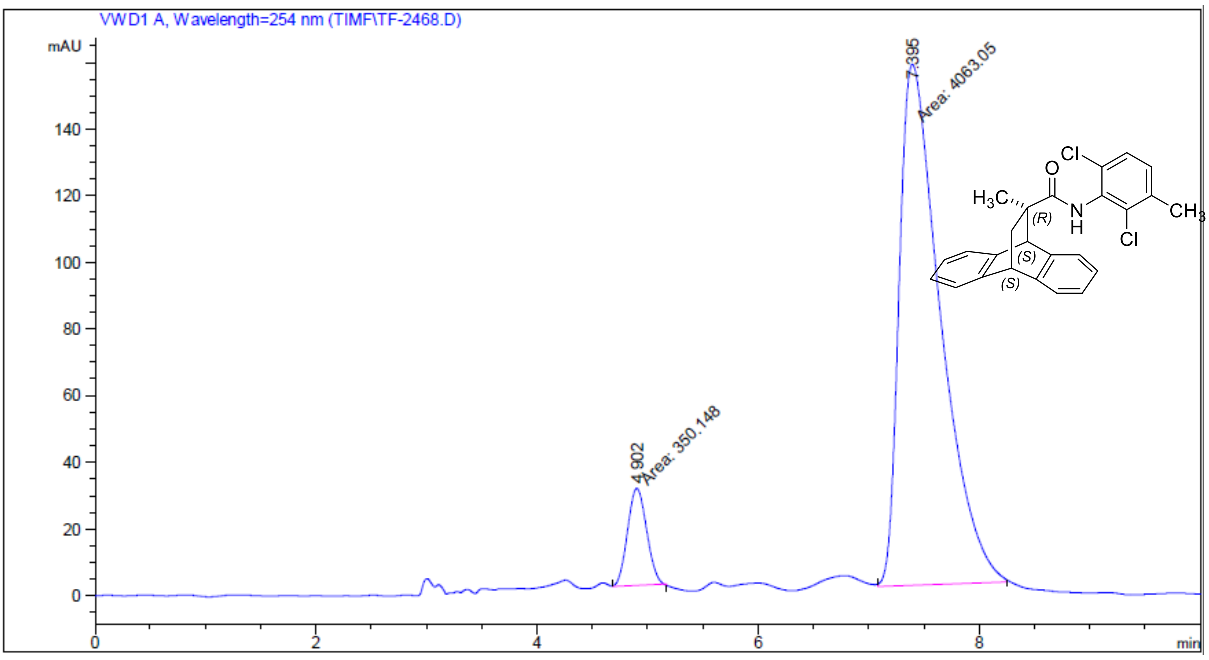

Figure S15. ^{1}H NMR spectrum for Compound (*R*)-2.

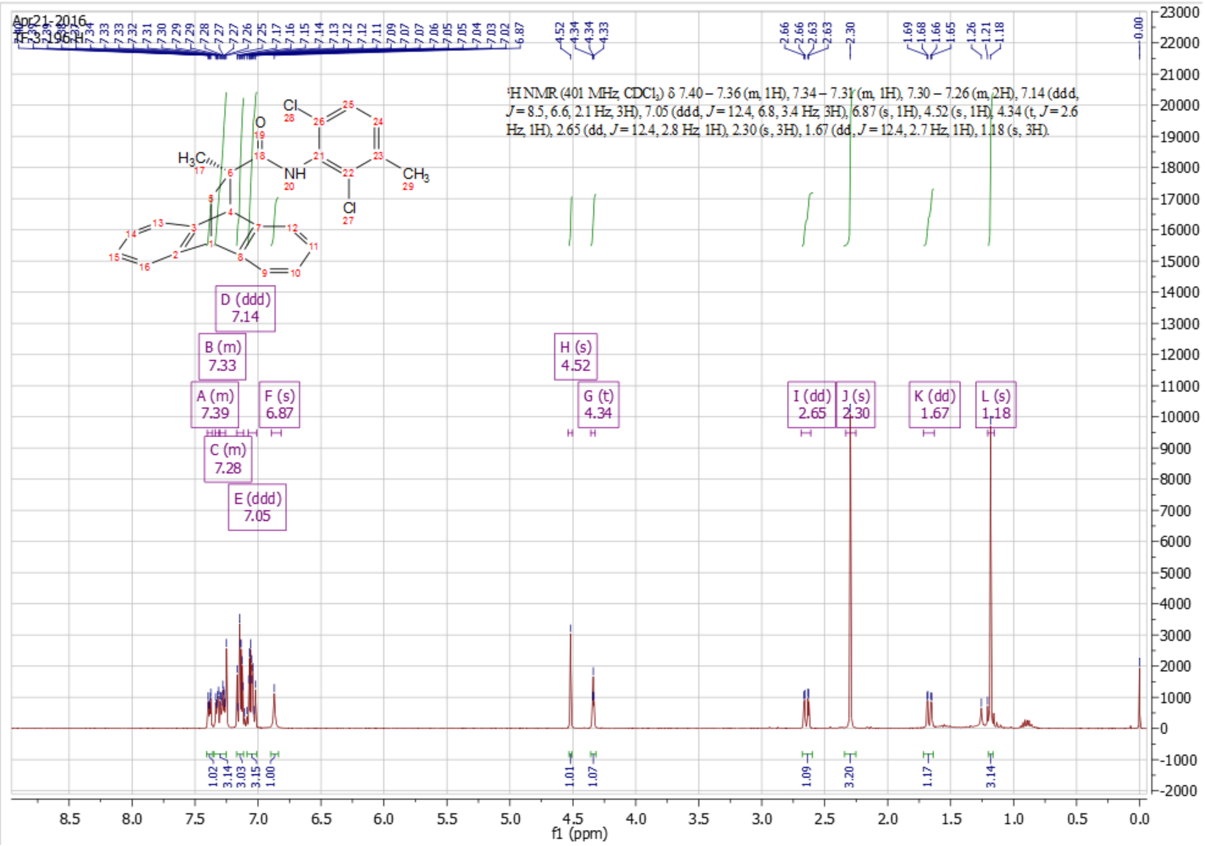

Figure S16. ^{13}C NMR spectrum for Compound (*R*)-2.

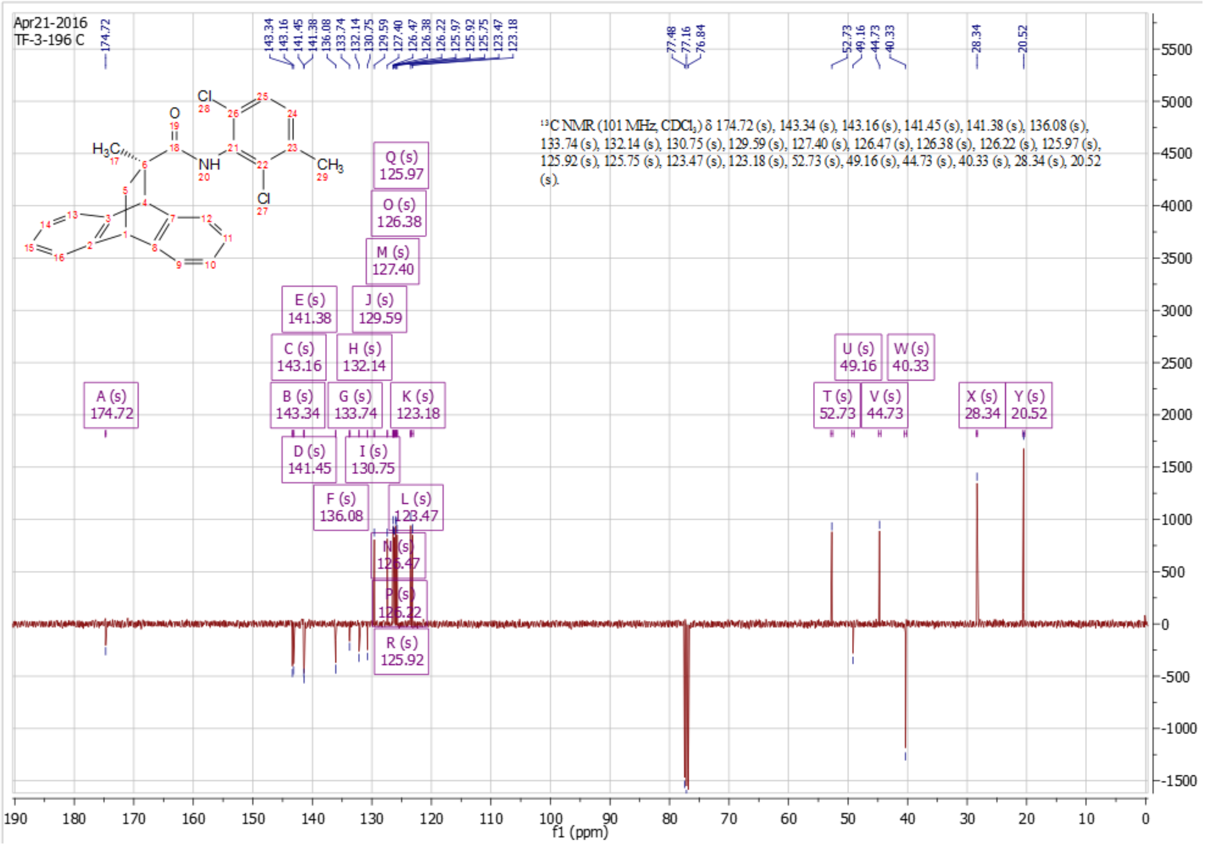

Supplement: Supplementary file 1 [file molecules-26-03799-s001.zip › molecules-1254912-supplementary.pdf]
